# Cold-Temperature Coding with Bursting and Spiking Based on TRP Channel Dynamics in *Drosophila* Larva Sensory Neurons

**DOI:** 10.3390/ijms241914638

**Published:** 2023-09-27

**Authors:** Natalia Maksymchuk, Akira Sakurai, Daniel N. Cox, Gennady S. Cymbalyuk

**Affiliations:** 1 Neuroscience Institute, Georgia State University, Atlanta, GA 30302-5030, USA; nmaksymchuk1@gsu.edu (N.M.); akira@gsu.edu (A.S.); dcox18@gsu.edu (D.N.C.); 2 Department of Biology, Georgia State University, Atlanta, GA 30302-5030, USA

**Keywords:** rate of temperature change, thermal sensation, computational modeling, cold nociception, biophysics of temperature coding

## Abstract

Temperature sensation involves thermosensitive TRP (thermoTRP) and non-TRP channels. *Drosophila* larval Class III (CIII) neurons serve as the primary cold nociceptors and express a suite of thermoTRP channels implicated in noxious cold sensation. How CIII neurons code temperature remains unclear. We combined computational and electrophysiological methods to address this question. In electrophysiological experiments, we identified two basic cold-evoked patterns of CIII neurons: bursting and spiking. In response to a fast temperature drop to noxious cold, CIII neurons distinctly mark different phases of the stimulus. Bursts frequently occurred along with the fast temperature drop, forming a peak in the spiking rate and likely coding the high rate of the temperature change. Single spikes dominated at a steady temperature and exhibited frequency adaptation following the peak. When temperature decreased slowly to the same value, mainly spiking activity was observed, with bursts occurring sporadically throughout the stimulation. The spike and the burst frequencies positively correlated with the rate of the temperature drop. Using a computational model, we explain the distinction in the occurrence of the two CIII cold-evoked patterns bursting and spiking using the dynamics of a thermoTRP current. A two-parameter activity map (Temperature, constant TRP current conductance) marks parameters that support silent, spiking, and bursting regimes. Projecting on the map the instantaneous TRP conductance, governed by activation and inactivation processes, reflects temperature coding responses as a path across silent, spiking, or bursting domains on the map. The map sheds light on how various parameter sets for TRP kinetics represent various types of cold-evoked responses. Together, our results indicate that bursting detects the high rate of temperature change, whereas tonic spiking could reflect both the rate of change and magnitude of steady cold temperature.

## 1. Introduction

Sensing cold temperatures is crucial for the survival of animals. To trigger physiological and behavioral protective responses, primary sensory neurons are capable of reliably detecting potentially harmful stimuli and reporting the rate of temperature change and the magnitude of cold temperature [1,2,3,4]. In *Drosophila* larva, Class III (CIII) primary sensory neurons have been shown to detect light mechanical touch [5,6] and noxious cold [7,8,9,10] stimuli. CIII neurons cover approximately 70% of each abdominal hemisegment of the *Drosophila* larva body wall [11]. In each hemisegment, there are five CIII neurons, and their dendrites tile the larval body wall across the dorsal, lateral, and ventral aspects [9,11]. These CIII neurons are distinct in both their morphology and function from three other classes of peripheral multidendritic sensory neurons—CI, CII, and CIV—innervating the epidermis of *Drosophila* larva [9,11,12,13]. The CI neurons are proprioceptors, the CII neurons are mechanosensors and thermosensors, and the CIV neurons are polymodal nociceptors that sense a variety of stimuli including chemicals, harmful heat, intense mechanical inputs, and ultraviolet light neurons [9,11,12,13].

We demonstrated that CIII neurons encode the rate of temperature decrease and the magnitude of cold temperature [14]. The majority of CIII neurons responded with a peak in the spiking rate, detecting a fast change of temperature and steady spiking activity at a stationary temperature, with the spiking rate corresponding to the temperature value. It is not well understood how different characteristics of the same stimulus, e.g., rate of temperature change and cold-temperature magnitude, can be reliably encoded by the same primary sensory neuron. For the *Drosophila* model system, a wealth of available neurogenetic, pharmacological, and electrophysiological tools are available to address this question [9,15,16,17,18,19].

Informed by feature coding in other sensory systems, we investigated temporal characteristics of thermal signals and classified qualitatively distinct events in patterns of CIII responses to cold stimulation. For example, bursting and spiking distinguish types of information in electric stimuli coding in electric fish, *Apteronotus leptorhynchus* [20], temperature coding in mammalian cold receptors [21,22,23,24], and mechanical stimuli in normal, inflammatory, and neuropathic conditions in mammalian high-threshold mechanoreceptors [25]. The importance of temporal properties of electrical activity (patterns) was shown in studying physiological versus pathological conditions such as mechanical and cold hypersensitivity and inflammatory conditions [25,26,27]. 

In this work, we combined electrophysiological and modeling approaches to investigate the cold-evoked activity patterns of CIII neurons during the transient and steady phases of cold-temperature stimulation. We identified different basic types of activity, spiking and bursting, and showed that different features of thermal information, such as the rate of temperature change and cold-temperature magnitude, are represented by distinct activities (spike-pattern code). We developed a computational model of CIII neurons that suggested that the interaction of TRP channels with a pattern-generating mechanism of the cell plays an important role in the encoding of specific features of the thermal signal. 

We developed and analyzed our biophysical model in two stages that correspond to two levels of model complexity. The level-I model represents TRP currents as a passive, lumped TRP current, i.e., a TRP leak current, to map basic steady stationary and oscillatory states under two parameters variation—temperature (Temp) and the TRP conductance (*G_LTRP_*)—and to match experimentally observed elements of spiking responses. For the level-II model, we included a TRP current with dynamical conductance governed by basic processes, temperature-dependent activation, and Ca^2+^-dependent inactivation, to reproduce transient characteristic CIII responses. With this approach, we investigated different temperature coding mechanisms depending on the kinetics of TRP currents and observed how TRP current dynamics shaped the trajectory of TRP current conductance and controlled activity pattern.

## 2. Results

### 2.1. Computational Model Activity Is Equipped to Reproduce Patterns of CIII Neurons

**Figure 1:**
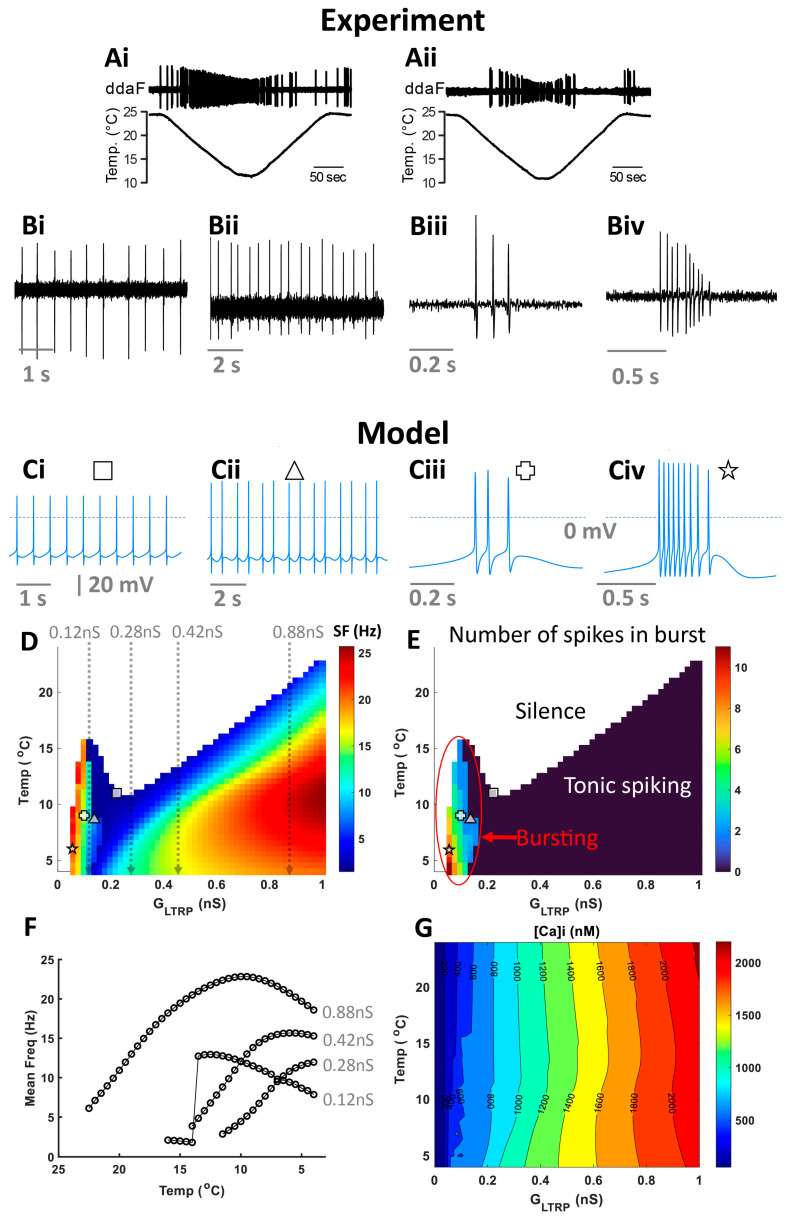
**Responses of CIII neurons to cold stimulation exhibited a wide repertoire of activity patterns.** (**A**) Two example recordings of spiking activity of CIII neurons responding to a slow temperature change. Spiking activities of CIII neurons were recorded as current pulses under voltage-clamp mode. We identified several basic types of CIII activity at different steady cold-temperature values: spiking (**Bi**), period-2 spiking (**Bii**), and bursting (**Biii**,**Biv**); (**Bi**,**Bii**) at 10 °C, (**Biii**) at 15 °C, and (**Biv**) at 17 °C. We found corresponding level-1 model activities (**Ci**–**Civ**) and marked them on two-parameter (*G_LTRP_*, Temperature) color maps of mean frequency (**D**) and the average number of spikes in a burst (**E**), with corresponding symbols (defined on the top of each activity panel). For bursting activity, the mean frequency was calculated as the mean intra-burst frequency (**D**). (**F**) firing rate versus temperature at different values of *G_LTRP_*: 0.12, 0.28, 0.42, and 0.88 nS. (**G**) Two-parameter (*G_LTRP_*, Temperature) contour map for intracellular Ca^2+^, [Ca^2+^]_i_.

By analyzing electrophysiological recordings (Figure 1(Ai,Aii,Bi–Biv)), we identified three types of activity that CIII neurons produced at steady or slowly changing cold temperatures: tonic spiking, period-2 spiking (doublets of action potentials), and bursting (Figure 1(Bi–Biv)). To understand possible mechanisms underlying these diverse cold-evoked CIII responses, we developed a computational model of the CIII neuron based on transcriptomic data obtained from *Drosophila* larva CIII cells [9,14]. First, we investigated the steady-state activity regimes obtained for the level-I model, which has constant *G_LTRP_* and temperature (Section 4). Our model reproduces the identified experimental events (Figure 1(Bi–Biv)) at certain values of temperature and *G_LTRP_*: tonic spiking (Figure 1(Ci)), period-2 spiking (Figure 1(Cii)), and bursting (Figure 1(Ciii,Civ)).

We generated a map that color-codes the mean frequency under the variation of two parameters: *G_LTRP,_* and temperature. Representative electrical activities at a given temperature (Temp) and *G_LTRP_* are shown in Figure 1(Ci–Civ) and are indicated on color maps with corresponding symbols (Figure 1D,E). The CIII model is silent at warmer temperatures and low values of *G_LTRP_* (white color in Figure 1D). In the case of temperature decrease, the model starts generating activity at a certain threshold temperature that is determined by *G_LTRP_* (shown with color in Figure 1D). We identified three areas of different major types of activity: silence, bursting, and tonic spiking (Figure 1E). Depending on temperature and *G_LTRP_*_,_ the CIII model has a different number of spikes in a burst (Figure 1E).

Next, we investigated the dependence of the spiking rate produced by the CIII model over temperature. In our previous work [14], we found high variability in the dependence of spiking rate over temperature (temperature-response curve) between individual CIII neurons. Some CIII cells showed a monotonic increase of spiking rate with temperature decrease, while there were also cells that had a maximum spiking rate at a certain cold temperature. We explained these differences between the temperature-response curves by variability in the parameters of TRP currents. With a simpler level-I CIII model, we consider an additional mechanism of possible variability in CIII neurons’ temperature-response curves (Figure 1F). We found that the character of the dependence of spiking rate over temperature can also be influenced by different levels of intracellular Ca^2+^ ([Ca^2+^]_i_), with the main Ca^2+^ influx produced by TRP and voltage-gated Ca^2+^ channels. In turn, [Ca^2+^]_i_ activates Ca^2+^-dependent K^+^ currents. The colormap (Figure 1G) shows how [Ca^2+^]_i_ changes with temperature and *G_LTRP_*. At *G_LTRP_* equal to 0.12 nS, the spiking rate initially increases to a maximal value and then slowly declines with temperature decrease (Figure 1F); with *G_LTRP_* increased to 0.28 nS, the firing rate monotonically increases with the temperature; at *G_LTRP_* equal to 0.42 nS, the spiking rate monotonically increases to saturation; at a higher value of *G_LTRP_* 0.88 nS, spiking rate has a peak at ~10 °C and decreases at lower temperatures. With the increase of TRP conductance, intracellular Ca^2+^ grows. Higher Ca^2+^ concentration, in turn, leads to a higher activation of calcium-activated K^+^ currents. 

### 2.2. Two Types of Cold-Evoked CIII Activity Patterns during Temperature Changes: Bursting and Tonic Spiking

**Figure 2:**
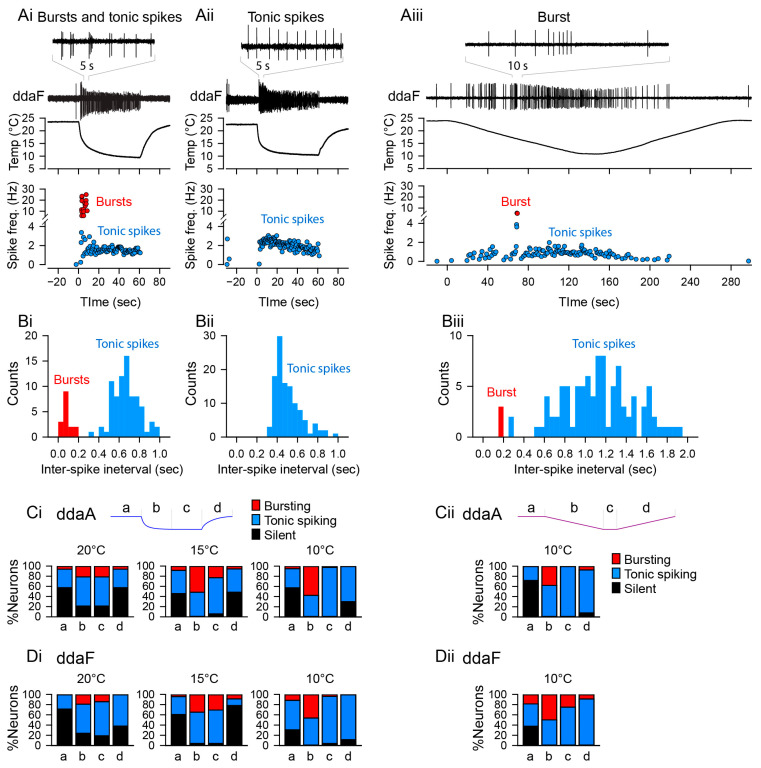
**Neuronal activity patterns: bursting and tonic spiking, the proportion of which changes depending on the stimulus temperature and time.** (**Ai**–**Aiii**) Representative data showing the patterns of spiking activity of CIII neurons evoked by the fast stimulation protocol (**Ai**,**Aii**) and the slow stimulation protocol (**Aiii**) decreasing the temperature down to 10 °C. Some neurons exhibited bursting activity (**Ai**,**Aiii**), while others exclusively displayed tonic spikes (**Aii**). Spiking activity of CIII neurons (ddaF, top), changes in saline temperature (middle), and plots of instantaneous spike frequencies (bottom) are shown. Spike groups with spike intervals of 0.2 s or less were defined as bursts (red), while those with larger spike intervals were defined as single spikes (blue). (**Bi**–**Biii**) Histograms of spike intervals derived from the respective neuron types (Bi and Biii, exhibiting bursting activity; Bii, displaying only tonic spikes) reveal distinct proportions of spike intervals for each type. (**Ci**,**Cii**,**Di**,**Dii**) In ddaA (**Ci**,**Cii**) and ddaF (**Di**,**Dii**), bar graphs depict the proportions of neurons exhibiting bursting activity versus those displaying only tonic spikes in response to both the fast stimulation protocols (**Ci**,**Di**) at three different temperatures (20 °C, 15 °C, and 10 °C) and the slow stimulation protocols (**Cii**,**Dii**). Each bar graph represents the % of the neurons exhibiting bursting acuztivity (red). Those showing only tonic spikes (blue), or those that are silent (black) at room temperature (a), during the falling phase (b), at a steady phase temperature (c), and during the rising phase (d) of the fast (**Ci**,**Di**) and slow stimulation protocols (**Cii**,**Dii**) are drawn schematically above the graphs.

In our previous study, we investigated the responses of two subtypes of CIII neurons (ddaA and ddaF) that encode the rate and magnitude of decrease in temperature; we found that their patterns of activities evaluated in terms of spiking rate were highly variable across the neurons [14]. Here, we report that based on the response spiking patterns in experiments, both ddaA and ddaF neurons exhibited two types of activities: bursting and tonic spiking (Figure 2A). With a fast temperature change from room temperature (Troom) down to 10 °C, the bursting responses were detected—as groups of spikes with interspike intervals smaller than 0.2 s (instantaneous spike frequency above 5 Hz, Figure 2(Ai), red symbols). In such neurons, the histogram of spike intervals had a peak below 0.2 s (Figure 2(Bi)). In contrast, the tonic spikers exhibited only spiking activity with interspike intervals larger than 0.2 s (Figure 2(Bii)). We found that approximately half of the CIII neurons responded with one or more bursts to a 60 s noxious cold-temperature stimulation (10 °C: 57.5% of ddaA, *n* = 23 of 40; 46.2% of ddaF, *n* = 12 of 26). The proportion of neurons that exhibited bursts declined as the target temperature became warmer; with 15 °C stimulation, 51.4% of ddaA (*n* = 18 of 35) and 39.1% of ddaF (*n* = 9 of 23) exhibited bursting, and with 20 °C stimulation, 24.2% of ddaA (*n* = 8 of 33) and 19.0% of ddaF (*n* = 4 of 21). The repeated stimulation of the same neurons did not induce significant changes in the characteristics of their spiking responses (Figure S2). 

The bursting activity of CIII neurons was not periodic but rather the rate of burst occurrence was constantly changing, especially during the fast stimulation protocol. Moreover, bursts were often mixed with single spikes between them. During the stimulus, the activity transits from bursting into mostly tonic spiking activity at a steady temperature level, whereas in a few cases, neurons kept bursting discharge during stationary cold temperatures as well. The proportion of neurons showing bursting activity is also time-dependent, appearing most frequently at the beginning of stimulation (phase b in Figure 2(Ci,Di)), decreasing during the steady phase of stimulation (phase c in Figure 2(Ci,Di)), and rarely seen during the post-stimulus (phase d in Figure 2(Ci,Di)), temperature-increase phase. With the 10 °C stimuli, 57.5% of ddaA neurons (*n* = 23 of 40) and 49% of ddaF neurons (*n* = 12 of 26) showed bursting activity during the first half (0–30 s) of the stimulus; but during the steady-state temperature (30–60 s), 2.5% of ddaA (*n* = 1 of 40) and 3.8% ddaF neurons (*n* = 1 of 26) exhibited bursts. No bursting activity was detected during the temperature increase in both ddaA and ddaF types of CIII neurons. With the 15 °C stimuli, 51.4% of ddaA neurons (*n* = 18 of 35) and 34.8% of ddaF neurons (*n* = 8 of 23) showed bursting activity during the first half (0–30 s) of the stimulus. During the steady-state temperature (30–60 s), 22.9% of ddaA (*n* = 8 of 35) and 30.4% of ddaF neurons (*n* = 7 of 23) showed bursts. During the temperature increase (60–90 s), the bursting activity was seen in 2.9% of ddaA (*n* = 1 of 35) and 8.7% ddaF neurons (*n* = 2 of 23). With the 20 °C stimuli, 21.2% of ddaA neurons (*n* = 7 of 33) and 19.0% of ddaF neurons (*n* = 4 of 21) showed bursting activity during the first half (0–30 s) of the stimulus. During the steady-state temperature (30–60 s), 21.2% of ddaA (*n* = 7 of 33) and 14.3% ddaF neurons (*n* = 3 of 21) exhibited bursting. During the temperature increase (60–90 s), 6.1% of ddaA (*n* = 2 of 33) but no ddaF neurons (*n* = 0 of 21) showed bursts.

A large proportion of the neurons exhibiting bursting activity had a pronounced peak in spike frequency immediately after the fast temperature drop (73.9% of ddaA, *n* = 17 of 23; 66.7% of ddaF, *n* = 8 of 12). In the previous study, we showed that a fast temperature stimulus of 10 °C produced a significant peak in spike frequency immediately after the temperature change in most of the cells (70% of ddaA and 61.5% of ddaF) [14]. These results indicate that the initial peak in spike frequency that occurs during fast temperature changes is shaped, at least in part, by bursting activity. 

The bursting activity was also seen during the slow stimulation protocol (Figure 2(Aiii,Biii)). Overall, 38.1% of ddaA (*n* = 8 of 21) and 56.3% ddaF (*n* = 9 of 16) exhibited bursting activity. The proportion was also dependent on the phase of stimulation (Figure 2(Cii,Dii)); during the downslope of temperature, bursting activity was observed in 38.1% of ddaA (*n* = 8 of 21) and 50% of ddaF (*n* = 8 of 16), while no ddaA (*n* = 0 of 21) and 25% of ddaF (*n* = 4 of 16) showed bursting at the steady-state temperature. During the rising temperature, 7.7% of ddaA (*n* = 1 of 13) and 18.2% of ddaF (*n* = 2 of 11) exhibited bursting. 

### 2.3. Bursts and Spikes in Response Patterns Differentiate Distinct Properties of Thermal Signals

**Figure 3:**
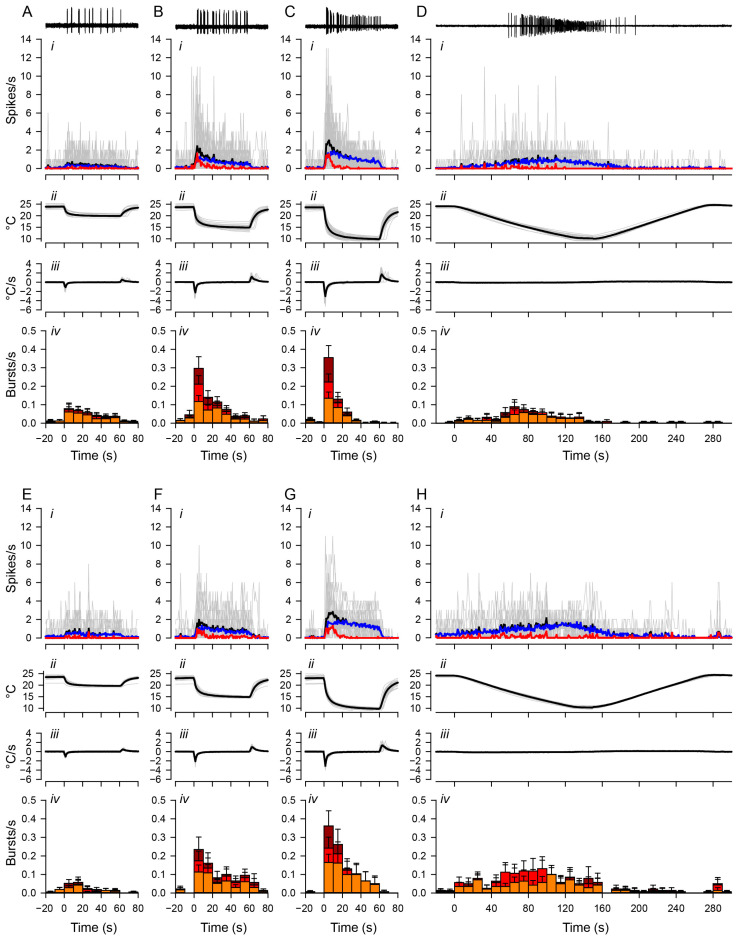
**Distinct temporal changes in bursting and tonic spiking.** Spiking activities of ddaA (**A**–**D**) and ddaF (**E**–**H**) in response to the fast stimulation protocol (20 °C, (**A**,**E**); 15 °C, (**B**,**F**); 10 °C, (**C**,**G**)) and the slow stimulation protocol (**D**,**H**). Representative traces of spiking responses to low-temperature stimuli are shown at the top. The panels below the traces show (**i**) the plot of averaged spiking rate (spikes/s) against time, (**ii**) the temperature (°C), (**iii**) the rate of temperature change (°C/s), and (**iv**) the bar graphs showing the rate of burst occurrences (bursts/s). Each bar shows the average rate of burst occurrence and is divided into three colors based on the number of spikes in the burst: orange, two-spike bursts; red, three-spike bursts; dark red, bursts with four or more spikes. In graphs (**i**), the grey traces show individual data; black traces show the mean values; blue traces show the mean values of tonic spikes; red traces show the mean values of bursting spikes. In these graphs, only spikes that lasted three or more at ISI intervals of 0.2 s or less were considered bursting spikes. The number of ddaA and ddaF neurons and the corresponding number of animals are as follows: (**A**) 33 ddaA neurons in 21 animals, (**B**) 35 ddaA neurons in 22 animals, (**C**) 40 ddaA neurons in 24 animals, (**D**) 21 ddaA neurons in 14 animals, (**E**) 21 ddaF neurons in 18 animals, (**F**) 23 ddaF neurons in 19 animals, (**G**), 26 ddaF neurons in 21 animals, (**H**) 16 ddaF neurons in 13 animals. Time zero is set to the onset of the stimulation. Error bars show standard errors.

The results described so far have shown that the peaks in the spiking rate occurred in association with the rapid temperature changes and that the bursts mostly occurred at the beginning of the temperature stimulus. To investigate how these two activity patterns are correlated with the rate of temperature change or/and the intensity of the cold stimulus, we analyzed the bursting activity of CIII neurons separately from tonic spiking activity using fast and slow temperature protocols (Figure 3).

Bursting activities were induced by both fast and slow stimulation protocols, and there was no significant difference in the number of bursts, given that the temperature drop had the same temperature difference. For ddaA neurons, the total number of bursts produced by the fast protocol of a 10 °C drop was 4.8 ± 0.76 (mean ± SEM), while the slow protocol produced 4.8 ± 1.5 (mean ± SEM) bursts. For ddaF neurons, the fast protocol produced 6.1 ± 1.4 bursts, while the slow protocol produced 6.3 ± 2.0 bursts. There was no statistical significance between the fast and slow stimulation protocols (ddaA, *p* = 0.91; ddaF, *p* = 0.92, both assessed by the Mann–Whitney Rank Sum test). Rather, we found that the rate and pattern of burst occurrences changed with the profile of stimulation. In response to the fast stimulation protocol with end temperatures of either 15 °C or 10 °C, the temporal distribution of burst occurrences became more skewed toward the early part of the stimulus due to an immediate decline of the frequency of the burst occurrence (red traces in Figure 3(Ai–Ci)). During the stimulation (from 0 to 60 s) at 15 °C and 10 °C, the rate of burst occurrences declined significantly in ddaA (Figure 3(Biv,Civ); 15 °C, *p* < 0.001; 10 °C, *p* < 0.001 by Friedman RM ANOVA on ranks, *n* = 40) and in ddaF (Figure 3(Fiv,Giv); 15 °C, *p* < 0.001; 10 °C, *p* < 0.001 by Friedman RM ANOVA on ranks, *n* = 25). In contrast, the rate of burst occurrences was kept relatively constant during 20 °C stimulation (Figure 3(Aiv,Eiv)). The peak frequency of burst occurrences in the first 10 s increased with increased magnitudes (20 °C, 15 °C, 10 °C) of the stimulation (ddaA, *p* < 0.001 by one-way RM ANOVA, *n* = 33–40; ddaF, *p* < 0.001 by one-way RM ANOVA, *n* = 21–25). These results suggest that the accumulation of peaks in the spiking rate in response to the rapid temperature change [14] is largely due to the concentrated bursting activity, whereas the tonic spiking is attributed largely to the steady-state responses during the stimulation.

In contrast to a fast stimulus, the slow stimulation protocol with the end temperature of 10 °C resulted in a broad distribution of bursting activity across all temperature ranges (Figure 3D,H). The bursting activity was also sparse, and the distribution of bursting activity was not concentrated in the same way as the distribution of peak activity. From room temperature to the bottom of the ramp (0 to 150 s), ddaA neurons showed a statistically significant difference in the median values of burst frequencies (*p* = 0.020 by Friedman RM ANOVA on ranks) but not in ddaF (*p* = 0.057 by Friedman RM ANOVA on ranks). In ddaA, all pairwise multiple comparison procedures (Tukey test) provided no statistical difference among different time bins (*p* > 0.05). 

**Figure 4:**
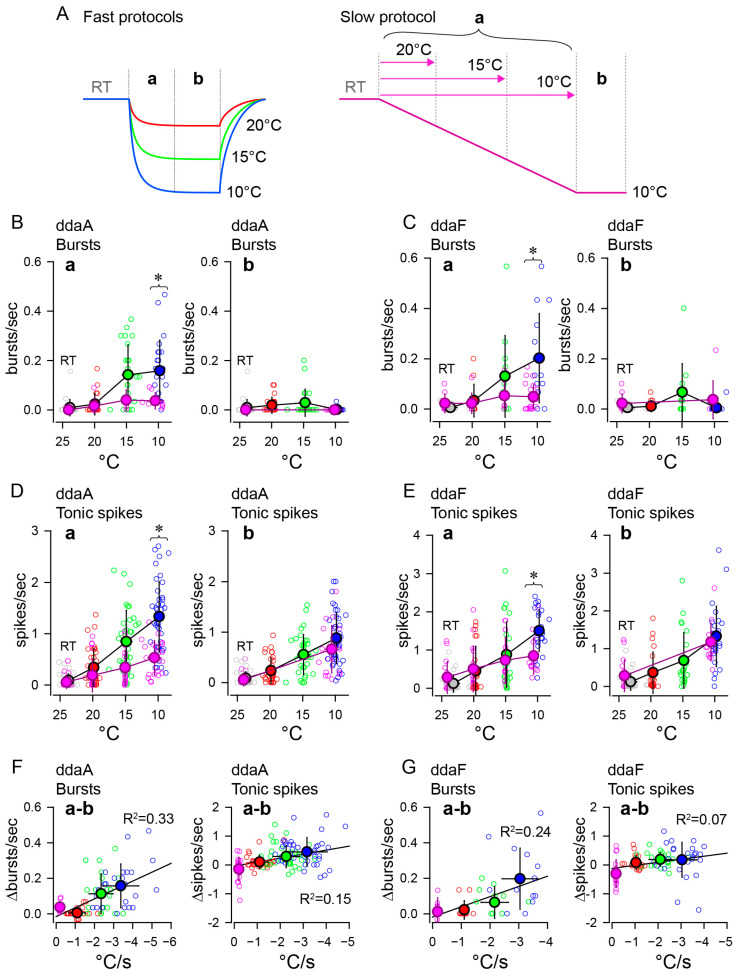
**Bursting and tonic spiking responded differently to the rate of change and magnitude of low-temperature stimulation**. (**A**) Schematic drawings illustrating the fast (left) and slow (right) stimulation protocols. In the fast protocol, the initial 30 s was designated as the temperature-drop period (**a**), followed by 30 s of steady state (**b**). For the slow stimulation protocol, the initial 120 s was designated as the temperature-drop period (**a**), followed by 30 s of steady state (**b**). In the slow protocol, bursting and spiking activities during the temperature-drop period (**a**) were recorded in three intervals: from the beginning of the temperature drop to 20 °C, 15 °C, and 10 °C, respectively. The line’s color indicates the temperature stimulus type: red for fast 20 °C, green for fast 15 °C, blue for fast 10 °C, and pink for slow 10 °C, aligning with the colors of symbols in the B-G graph. (**B**,**C**), The average rate of burst occurrence ((**B**), ddaA; (**C**), ddaF) was plotted against the target temperatures during the initial 30 s or 120 s of the temperature drop (**a**) after the onset of stimulation and the following 30 s (**b**). (**D**,**E**), The average tonic spike frequency ((**D**), ddaA; (**E**), ddaF) plotted against the target temperatures as in (**B**,**C**). For all graphs, the dataset is the same as the one used in Figure 3, but only data from neurons that exhibited bursting with more than three spikes per burst were included in the plot. The open circles are individual data whose colors indicate the target temperature of the stimulation (red, fast 20 °C; green, fast 15 °C; blue, fast 10 °C; pink, slow 10 °C). Gray symbols represent data at room temperature. The bold symbols filled with the same colors are the averages (mean ± SD). In some cases, horizontal error bars are too small to be hidden by symbols. (**F**,**G**), Changes in bursting or tonic spikes versus the rate of temperature change. The differences in the rate of burst occurrences (left) or the tonic spike frequency (right) between the initial temperature drop period and the steady-state period were plotted against the rate of changes in temperature. The lines in the (**a**,**b**) graphs show linear regression (see the text). Asterisks in the graphs indicate significant differences between the fast and slow protocols (see text).

To further compare the effects of different rates of change in temperature, we investigated the rates of burst occurrences and tonic spikes within two different time windows (initial falling phase and subsequent steady-state phase, c.f. Figure 4A) during the fast and slow stimulation protocols (Figure 4). In the fast stimulation protocols of three different amplitudes, the rate of burst occurrences during the initial falling phase (a) increased with the lowering of the target temperatures, reaching the maximal with the 10 °C stimuli (blue) in both ddaA (Figure 4(Ba)) and ddaF (Figure 4(Ca)). The increases were significant (ddaA, *n* = 21–24, *p* < 0.001 by one-way RM ANOVA; ddaF, *n* = 12–14, *p* < 0.001 by one-way RM ANOVA *p* < 0.001). Similar temperature-dependent increases in the occurrence of bursts were seen during the slow stimulation protocol (in ddaA, *n* = 8, *p* = 0.006 by one-way RM ANOVA, in ddaF, *n* = 9, *p* = 0.035 by one-way RM ANOVA). However, the rate of burst occurrences was lower on average and maxed out around 15 °C and then slightly declined, so it did not show the maximal response near 10 °C (pink symbols in Figure 4(Ba,Ca)). There were significant differences between fast and slow stimuli in burst frequency during the decreasing phase using the 10 °C stimulus (blue vs. pink; in ddaA, *p* = 0.001 by Mann–Whitney Rank Sum test; in ddaF, *p* = 0.02 by two-tailed *t*-test).

In contrast, during the subsequent steady-state temperature period in the fast protocol, the rate of burst occurrences did not increase significantly with temperature and was lower on average at 10 °C than at 15 °C (Figure 4(Bb,Cb); in the fast protocol on ddaA, *n* = 21–24, *p* = 0.020 by one-way RM ANOVA with the Holm–Sidak method; in the fast protocol on ddaF, *n* = 12–14, *p* = 0.025 by one-way RM ANOVA with the Holm–Sidak method). In the slow protocol, the rate of burst occurrences during the steady-state period did not differ significantly from that in the pre-stimulus period (ddaA, *p* =1.0; ddaF, *p* = 0.40 by one-way RM ANOVA). There was no statistical difference in steady states at 10 °C between the fast and the slow protocol (ddaA, *p* = 0.61; ddaF, *p* = 0.054, both by Mann–Whitney Rank Sum test). Taking into account the results from both the fast and slow protocols during two distinct periods (a and b), there were strong correlations between the changes in the rate of burst occurrence (Δbursts/s) and the rates of temperature drop (°C/s). This was evident when plotting the differences in burst occurrence rates between the two stimulus phases (a–b) against the rate of temperature drop during period a (Figure 4F,G, left graphs), with all having significant correlations obtained by Pearson correlation (Figure 4F left, ddaA bursts, *p* < 0.001, *n* = 32; Figure 4G left, ddaF bursts, *p* = 0.007, *n* = 23). These findings indicate that a higher initial rate of burst occurrences correlates to a higher rate of temperature change.

Tonic spike frequency increased with lowering the target temperatures during the initial falling phase (a) in both fast and slow protocols on both types of neurons (Figure 4(Da,Ea)). The increases were significant in all cases (*p* < 0.001, all by one-way RM ANOVA) in the fast protocol on ddaA (*n* = 33–40, *p* < 0.001) and ddaF (*n* = 21–26, *p* < 0.001), and in the slow protocol on ddaA (*n* = 21, *p* < 0.001) and on ddaF (*n* = 16, *p* < 0.001). In both types of neurons, the fast protocol (blue) elicited tonic spiking at higher frequencies compared to the slow protocol (pink) at 10 °C. (ddaA, *p* = 0.006 by Student’s *t*-test; ddaF, *p* = 0.023 by Mann–Whitney Rank Sum test). Unlike bursting, tonic spiking during the steady-state temperature exhibited a temperature-dependent increase in frequency (Figure 4(Db,Eb)). In the fast stimulation protocol, the steady-state (b) tonic spike frequency increased as the target temperature decreased in both ddaA and ddaF (red, green, and blue symbols in Figure 4(Db,Eb); *p* < 0.001 for both ddaA and ddaF by one-way RM ANOVA). Similar increases were seen in the slow stimulation protocol (ddaA, *p* < 0.001 by Wilcoxon Signed Rank test; ddaF, *p* < 0.001 by paired *t*-test), in which the tonic spike frequency during the 10 °C period was similar to that of the fast stimulation protocol (pink symbols). There was no statistical difference in steady states at 10 °C between the fast and the slow protocol (ddaA, *p* = 0.10 by Student’s *t*-test; ddaF, *p* = 0.41 by Mann–Whitney Rank Sum test). The results from both the fast and slow protocols during two distinct periods (a and b) revealed a correlation between the changes in the tonic spike frequency (Δspikes/s) and the rates of temperature change (°C/s). The correlation between the differences in the tonic spike frequency and the rate of temperature drop (Figure 4F,G, right graphs) is weak but statistically significant in both ddaA and ddaF neurons (Figure 4F, ddaA tonic spikes, *p* < 0.001, *n* = 129; Figure 4G, ddaF tonic spikes, *p* = 0.012, *n* = 86, both by Pearson correlation). This indicates that, as with the rate of burst occurrence, the faster the temperature change, the higher the frequency of tonic spikes.

**Figure 5:**
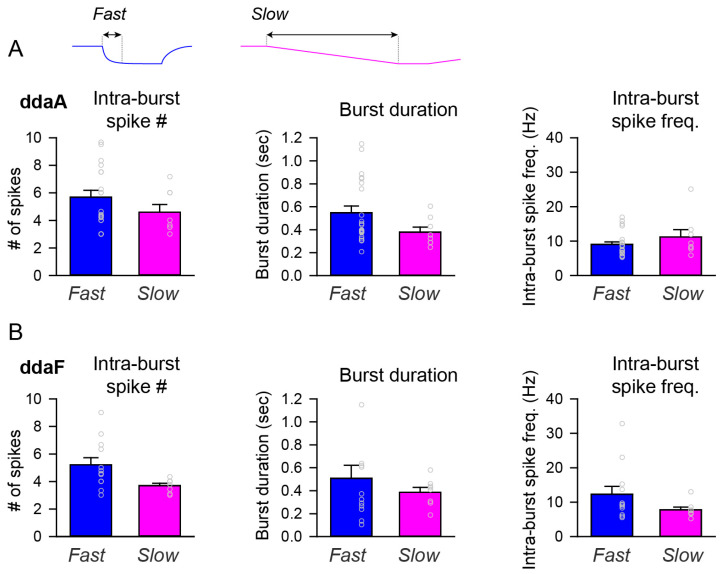
**Bar graphs comparing the burst parameters between fast and slow stimulation protocols.** (**A**,**B**) Comparisons were made between the fast and slow stimulation protocols on the averages of intra-burst spike numbers (left), burst duration (middle), and intra-burst spike frequency (right) for ddaA (**A**) and ddaF (**B**). The dataset is the same as the one used in Figure 3. There was a statistically significant difference in the number of intra-burst spikes in ddaF between the fast and slow stimuli ((**B**), Intra-burst spike #, *p* = 0.03 by Student’s *t*-test). Otherwise, no statistically significant differences were found in the number of intra-burst spikes in ddaA (*p* = 0.20 by Mann–Whitney Rank Sum test), the duration of a burst (ddaA, *p* = 0.10; ddaF, *p* = 0.97 by Mann–Whitney Rank Sum test), or the intra-burst spike frequency (ddaA, *p* = 0.36; ddaF, *p* = 0.11 by Mann–Whitney Rank Sum test).

The parameters of bursts, such as the intra-burst spike numbers, burst duration, and intra-burst spike frequency, varied largely across individual neurons. In ddaF, the fast stimulation protocol produced a larger number of intra-burst spikes on average than the slow stimulation protocol (*p* = 0.03 by Student’s *t*-test). Otherwise, there was no statistical difference in those parameters between the fast and slow stimulation protocols (Figure 5).

Altogether, CIII neurons responded specifically to the rate of temperature change and the cold-temperature magnitude by generating both burst and tonic spiking. Burst activity appears more frequently at the onset of a fast and large temperature drop and is highly skewed in its distribution to the onset of change. In contrast, they appear irregularly and in a widely distributed manner during mild cold stimuli and slow temperature drops. Bursting ceased to occur with sustained low temperature, while tonic spikes persisted in a magnitude-dependent manner. These results indicate that the slow temperature change and its magnitude were reflected mainly in the tonic spiking activity of CIII neurons, as described in the previous study [14], and that the bursting activity encodes the rate of change in temperature, although it could still occur when the temperature changed slowly.

### 2.4. Basic Mechanisms Distinguishing Neurons Exhibiting Bursts from Those with Only Tonic Spikes during Temperature Changes: Computational Modeling

**Figure 6:**
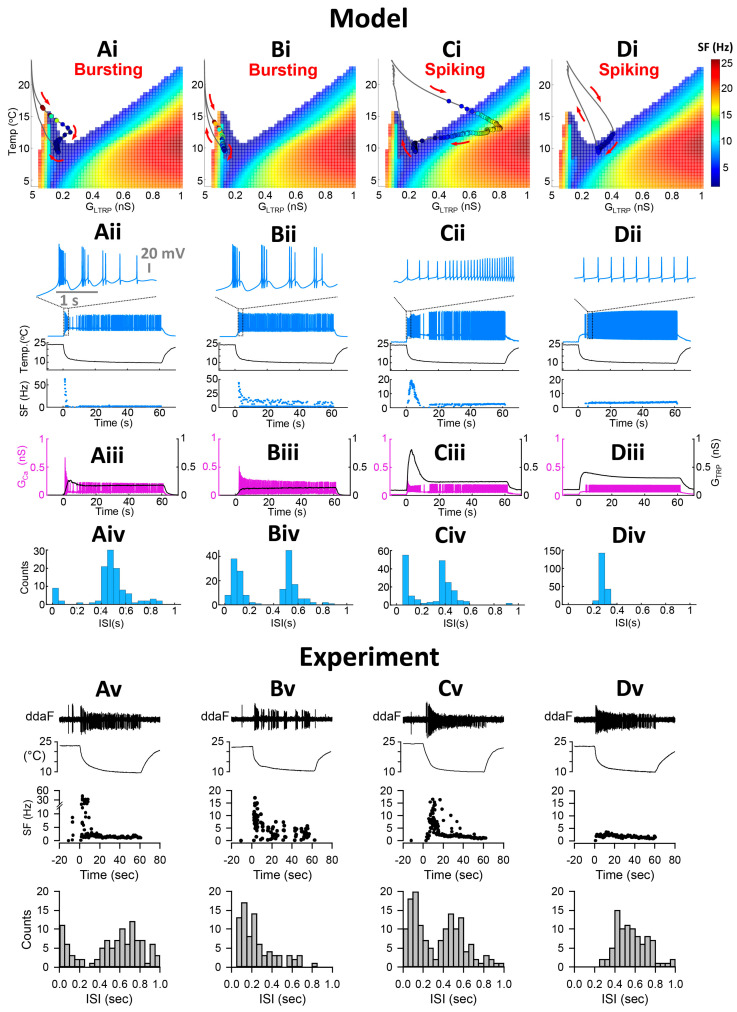
**Dynamics of the TRP current conductance control activity pattern.** (**Ai**–**Di**) Color maps of level-I model activity superimposed with trajectories of TRP current conductance of the level-II (full) model. Circles on the trajectory indicate individual spikes. Colors inside circles encode spike frequency. Red arrows indicate the direction of trajectory passage. (**Aii**–**Dii**) Full model electrical activity and instantaneous spike frequency (SF) of full CIII model in response to the fast stimulation temperature protocol. Insets show electrical activity patterns during the fast temperature drop. (**Aiii**–**Diii**) Conductances of TRP current and VGCC over time: *G_TRP_* and *G_Ca_*, respectively. (**Aiv**–**Div**) Model distributions of inter-spike intervals of the full CIII model under the fast stimulation protocol. (**Av**–**Dv**) Experimental extracellular electrical activities of CIII neurons, instantaneous frequency, and distribution of interspike intervals under the fast stimulation protocol. (**A**,**B**) represent neurons with bursting activity; (**C**,**D**) represent those with only tonic spikes; (**A**) initial bursting response during temperature change, then tonic spiking at a steady temperature; (**B**) there is a bursting activity during temperature change and at a steady temperature. (**C**) Initial transient high-frequency spiking activity and rapid adaptation. In the case of (**D**), after entering the spiking area of the map, the trajectory follows along with the border of the spiking area producing the same frequency. On the way back, model neurons are silent in all cases (**A**–**C**), which is consistent with experimental results. Model parameters for (**A**) $\bar{G_{TRP}}$ = 2 nS, *A* = 0.5 K^−1^, *N* = 5, *Th* = 10 °C; *Ca_h_* = 500 nM, $\tau_{hTRP}$ = 5 s; parameters for (**B**) $\bar{G_{TRP}}$ = 1.5 nS, *A* = 0.5 K^−1^, *N* = 5, *Th* = 8 °C; *Ca_h_* = 500 nM, $\tau_{hTRP}$ = 5 s; Parameters for (**C**) $\bar{G_{TRP}}$ = 6 nS, *A* = 0.3 K^−1^, *N* = 5, *Th* = 11 °C; *Ca_h_* = 500 nM, $\tau_{hTRP}$ = 5 s; Parameters for (**D**) $\bar{G_{TRP}}$ = 1.5 nS, *A* = 0.25 K^−1^, *N* = 1, *Th* = 17.5 °C; *Ca_h_* = 300 nM, $\tau_{hTRP}$ = 15 s; Circles on the trajectory indicate individual spikes. Color inside the circle encodes spike frequency. (**Av**–**Dv**) Experimental electrical activities and distribution of inter-spike intervals.

The level-I CIII model (Figure 1) describes the steady-state activity of a CIII neuron at different steady temperatures and the constant TRP conductance and, thus, does not describe transient CIII responses to temperature change with time. To investigate transient bursting or tonic spiking responses during the temperature change, we employed our full CIII model (level-II model) (Section 4). The full model has the TRP current conductance gated by temperature-dependent activation and Ca^2+^-dependent inactivation state variables [14]. These dynamics introduce a phasic-tonic response of the CIII model to cold-temperature stimulation [14]. Next, we projected the trajectory for instantaneous TRP conductance obtained with the level-II model onto the two-parameter map of the level-I model (Figure S1). This approach allows us to investigate how TRP current dynamics affect patterns of CIII activity. Our model shows that the dynamics of the TRP current can shape the trajectory of TRP conductance and produce various types of activity patterns with the same fast-temperature stimulation, reproducing the variability of experimental results: bursting activity during the fast temperature change, bursting activity during both temperature change and steady noxious cold temperature, tonic spiking activity with frequency peak during temperature change, and tonic spiking activity without peak in frequency (Figure 6). Depending on the parameters of the TRP current, the trajectory of the TRP instantaneous conductance passes through different areas of activity patterns on the color map, bursting or/and tonic spiking (Figure 6(Ai–Di)). On the left (Figure 6(Ai)), the trajectory passes through the bursting domain in the peninsula-like area and tonic spiking one; thus, we record bursting activity during temperature change, spiking activity at steady temperatures, and suppressed activity when temperature returns to the initial value (Figure 6(Aii)). Also, during temperature change, the trajectory closely approaches the bursting peninsula area that causes corresponding bursting activity (Figure 6(Ai,Aii)). This type of bursting relates to a response of an excitability type-3 neuron to a fast-changing stimulus [28,29]—the temperature in our case. In Figure 6(Bi,Bii), the trajectory totally passes through the bursting area of the peninsula, which reflects on model activity that shows bursting activity during both temperature change and steady-state temperature. Figure 6(Ci,Cii,Di,Dii) demonstrates that a neuron can have only tonic spiking activity if the trajectory passes through the tonic spiking area. In the case of Figure 6(Ci), the trajectory enters the high-frequency area during the temperature decrease, and therefore the CIII model response exhibits a peak in the spiking rate. In contrast, when the trajectory goes through the low-frequency spiking area, the model response does not exhibit a peak in the spiking rate (Figure 6(Dii)). Thus, considering variability in the parameters of the TRP current, we can obtain a variety of possible model electrical activity regimes that are consistent with experimental ones (Figure 6(Av–Dv)). 

Figure 6(Aiii–Diii) also shows that the parameters of the TRP current affect the balance between the TRP current and VGCC, which is important for activity patterns. At larger *G_Ca_* and smaller TRP conductance levels, we observe a bursting pattern (Aiii–Biii). When the balance between these currents is shifted towards a larger contribution of the TRP current than VGCC, the CIII neuron model produced tonic spiking activity (Aiii–Biii). 

Our model shows that the neurons exhibiting bursts have bimodal interspike interval (ISI) distribution with small ISIs (<~0.2 s) (Figure 6(Aiv–Biv)), which is consistent with experimental data (Figure 2(Bi) and Figure 6(Av)). Short ISIs correspond to bursting activity, whereas larger ISIs represent tonic spikes and pauses (Figure 6(Av–Cv)). Neurons that did not exhibit bursts, e.g., spiking neurons, have a mainly unimodal distribution of ISI with larger ISIs (Figure 6(Div,Dv)). Some neurons can respond with high-frequency spiking to temperature change (Figure 6(Cii)). Such activity has bimodal ISI distribution and can be also considered as a spiking neuron or a bursting neuron, which has one burst with a large number of intra-burst spikes that appear in the ISI distribution with short ISIs (Figure 6(Civ)). Experimental data confirm the existence of this pattern in CIII neurons (Figure 6(Cv)). 

About half of biological CIII neurons, 57.5% of ddaA, and 49% of ddaF, respond with bursting activity to fast temperature change from room temperature to 10 °C (Figure 2(Ci,Di)), whereas the rest of the neurons responded with tonic spiking activity. We propose that such variability in experimental CIII responses can be explained by variability in the expression of TRP channels, which have different properties. In terms of the computational model, such properties can be tested using the biophysical parameters of a TRP current. In order to validate the proposed assumption and mechanism proposed by the model, sets of three parameters of the TRP current, estimated by curve-fitting multiple temperature-response (spiking rate) curves obtained through experimentation in our previous work [14], were introduced to the model. The parameters assessed by the curve-fitting of spiking rates are as follows: temperature of the half-activation, *T_h_*, scaling factor for maximal TRP conductance, *B*, and the steepness of temperature dependence of TRP activation, *A*. Hence, we obtained bursting or spiking activity during temperature change depending on TRP parameter set. Representative examples of such activity are presented in Figure S3. As a result, 47.3% of model neurons responded with bursting activity and 42.8% with tonic spiking, which is consistent with experimental results. In addition, among the bursting neurons, 4.5% neurons showed plateau-like responses. The rest of the neurons exhibited a depolarization block (9.3%) and/or did not show a response during the first 30 s after stimulation (0.6%). 

Our results suggest that the dynamics of the TRP current explain the appearance of bursting and tonic activity patterns of CIII neurons under cold-temperature stimulation. 

### 2.5. CIII Activity Pattern during Fast Temperature Change Is Determined by the Rate of Temperature Decrease but Not the Cold-Temperature Magnitude

**Figure 7:**
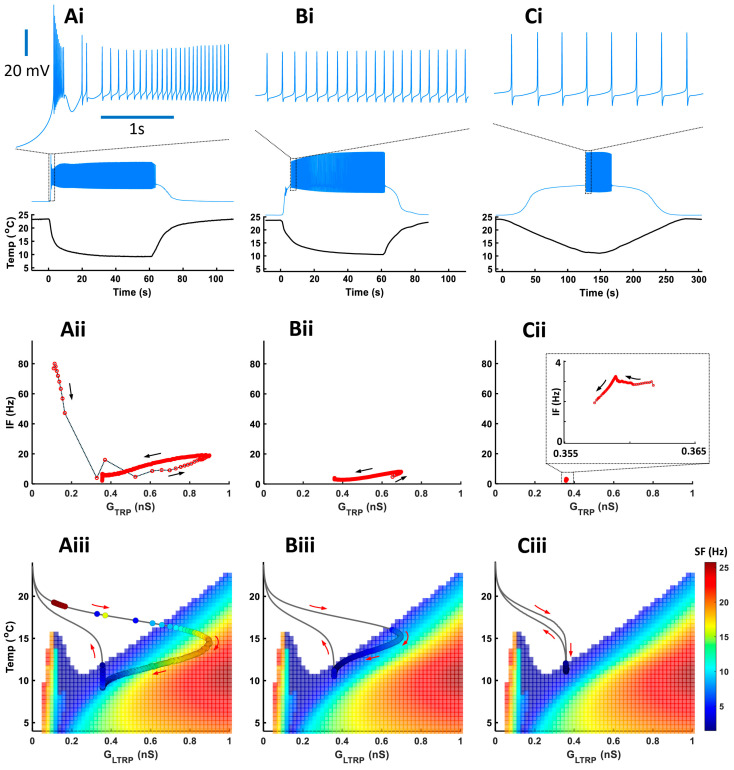
**The rate of temperature change affects activity patterns during a temperature drop (experimental temperature protocols).** (**Ai**–**Ci**) Model responses to three experimental temperature protocols with different rates of temperature change and the same minimal cold temperature of 10 °C. On the top are insets of electrical activity during a temperature drop, scale—3 s. (**Aii**–**Cii**) Instantaneous frequency versus TRP conductance. (**Aiii**–**Ciii**) Color maps of level-I model activity superimposed with trajectories of TRP current conductances of the level-II (full) model. Circles on the trajectory indicate individual spikes.

**Figure 8:**
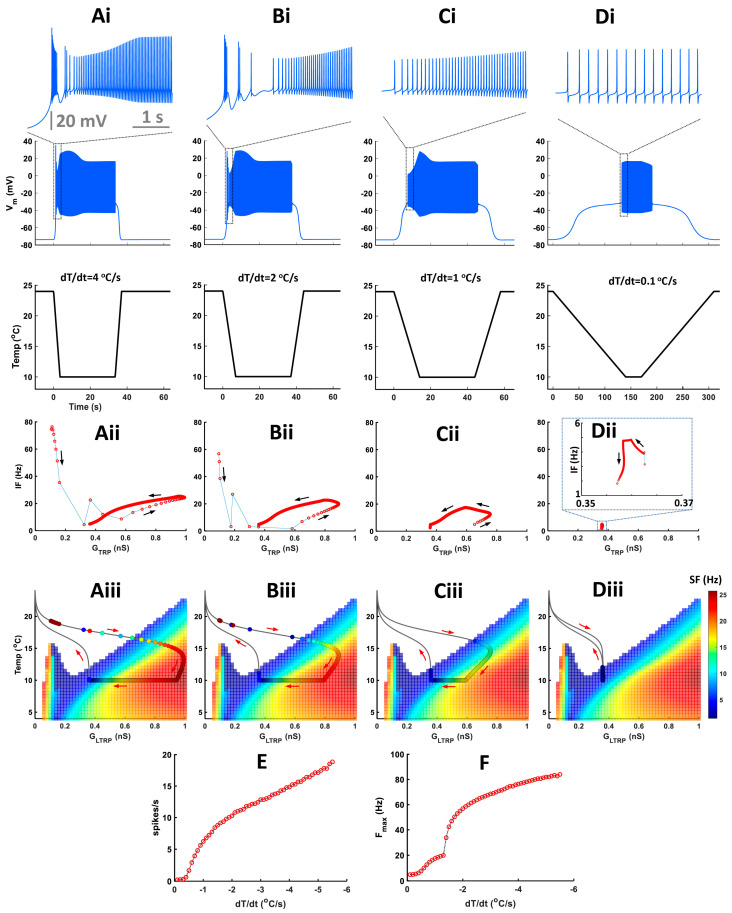
**The rate of temperature change affects activity patterns during a temperature decrease following trapezoid temperature protocols.** (**Ai**–**Di**) Model responses to trapezoid temperature protocols with different rates of temperature change and the same minimal cold-temperature values and corresponding model responses to these protocols. On the top are insets of electrical activity during a temperature drop, scale—5 s. (**Aii**–**Dii**) Instantaneous frequency versus TRP conductance. (**Aiii**–**Diii**) Color maps of level-I model activity superimposed with trajectories of TRP current conductances of the level-II (full) model. Circles on the trajectory indicate individual spikes. Color inside the circle encodes spike frequency. (**E**) Spiking rate during temperature change versus the rate of temperature change. (**F**) Maximal frequency during temperature change versus the rate of temperature change.

Experimental data showed that bursts mostly appeared during a fast temperature decrease. To investigate how different rates of temperature decrease affect the CIII activity pattern, we applied experimental fast and slow stimulation protocols with temperature decrease to 10 °C to our model (Figure 7). In fast stimulation protocols, the temperature decreases exponentially with time and the rate of temperature change, *dT/dt*, is different at every moment of time. To simplify the analysis of temporal properties of CIII activity, we also applied a trapezoid temperature protocol where temperature decreases and increases with a constant rate, *dT/dt* (Figure 8). Both exponential and trapezoid temperature protocols show that the rate of temperature change affects the electrical activity pattern of the CIII model: higher rates evoke bursting activity (Figure 7(Ai) and Figure 8(Ai,Bi)) whereas slower rates elicit tonic spiking activity (Figure 7(Bi,Ci) and Figure 8(Ci,Di)).

We built our model assuming that spike frequency is roughly proportional to TRP conductance, *G_TRP_*. This is the case when a neuron produces tonic spiking activity (regions with linear dependence in Figure 7(Aii,Bii) and Figure 8(Aii–Cii)). Bursting activity appears as a high-frequency cluster during a fast temperature change (Figure 7(Aii) and Figure 8(Aii,Bii)) which corresponds to a peak in the firing rate during a temperature change. Such bursting activity during a fast temperature change appears near the peninsula area due to the voltage-gated Ca^2+^ current (Figure 7(Aiii) and Figure 8(Aiii,Biii)). During a slow temperature change (Figure 7(Bii,Cii) and Figure 8(Cii,Dii)), we do not observe these clusters of bursts. When the temperature changes rapidly, TRP conductance, *G_TRP_*, reaches higher values, which is followed by spike frequency (Figure 7 and Figure 8(Aii)). This finding corresponds to a longer path of the trajectory of TRP conductance that reaches the high-frequency area of the two-parameter color map during a fast temperature change (Figure 7 and Figure 8(Aiii). In contrast, during a slow temperature change, we observe a small change in both TRP conductance and frequency as well as a shorter path of TRP conductance, despite the same temperature range (Figure 7(Bii,Biii,Cii,Ciii) and Figure 8(Cii,Ciii,Dii,Diii)). 

Higher rates of temperature change increase the spiking rate (Figure 8E) and maximal instantaneous frequency (Figure 8F). After increasing the rate of temperature change to a certain value (|*dT/dt*| > 1.3 °C/s in our case), we observe a transition from tonic spiking to bursting (Figure 8F). 

**Figure 9:**
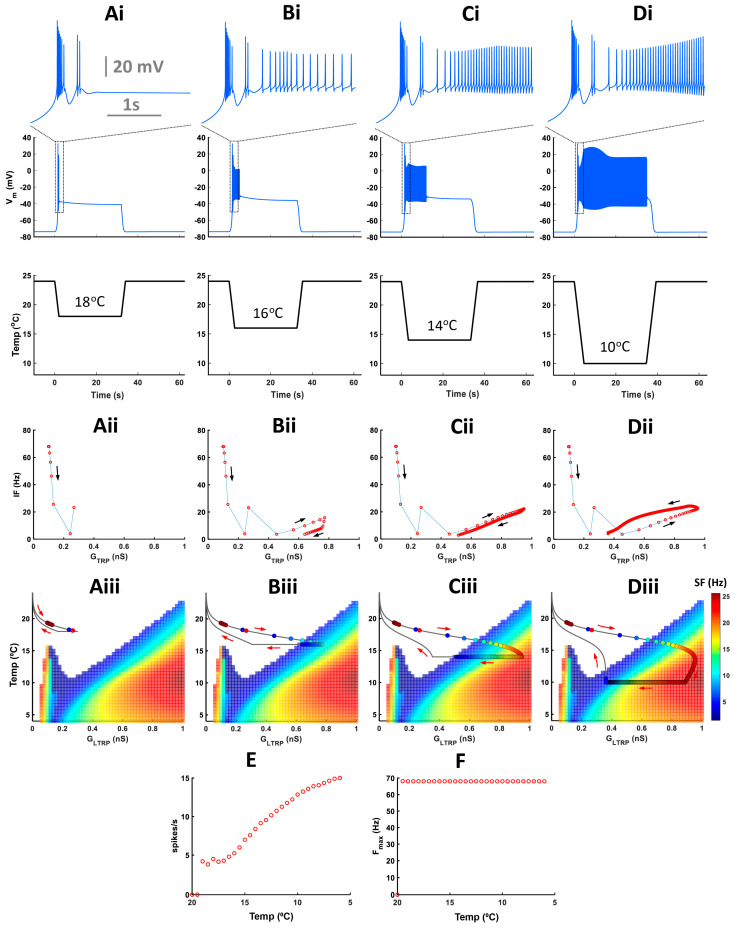
**The temperature magnitude does not affect activity patterns during a temperature drop**. (**Ai**–**Di**) Model responses to trapezoid temperature protocols with different cold-temperature magnitudes and constant rate of temperature change. On the top, there are insets of electrical activity during a temperature drop. (**Aii**–**Dii**) Instantaneous frequency versus TRP conductance. (**Aiii**–**Diii**) Color maps of level-I model activity superimposed with trajectories of TRP current conductances of the level-II (full) model. Circles on the trajectory indicate individual spikes. Colors inside circles encode spike frequency. (**E**) Spiking rate versus cold-temperature magnitude. (**F**) Maximal instantaneous frequency during temperature change versus cold-temperature magnitude.

Next, we investigated how cold-temperature magnitude affects CIII activity patterns. We applied a trapezoid temperature protocol with a fixed rate of temperature change and different minimal cold temperatures (Figure 9).

Cold-temperature magnitude does not affect activity patterns during temperature change but impacts activity only at steady-state cold temperatures (Figure 9(Ai–Di)). With lower temperatures, the instantaneous frequency of tonic spikes and spiking rate increases, but the maximal intra-burst frequency remains unchanged (Figure 9(Aii–Dii,E,F)). Also, with increasing low-temperature magnitude, the trajectory of instantaneous TRP conductance passes via a longer path reaching the high-frequency spiking area (Figure 9(Aiii–Diii)). 

## 3. Discussion

Growing evidence underscores the significance of spiking patterns in the responses of sensory neurons. This research highlights the functional role of bursting activity in enabling specialized roles that extend beyond the limitations of spiking-rate coding [20,23,30,31,32,33,34,35,36,37,38,39,40,41,42,43]. We investigated patterns within the activity of the primary sensory CIII neurons of *Drosophila* larvae, including two subtypes of these neurons: ddaA and ddaF. They generate transient spiking activity in response to temperature decreases, with a peak marking fast temperature change [14]. Here, we distinguished three basic patterns in their responses: tonic spiking, period-2 spiking, and bursting. We investigated the relationship between the occurrence of bursts and two aspects of the temperature change process: the rate of temperature change and the magnitude of the final temperature. The fraction of CIII neurons exhibiting bursts showed a dependence on the rate of temperature change. As the rate increased, the percentage of neurons that exhibited bursting activity also grew, ranging from 20% to 55%. Both the rate of burst occurrence and the tonic spike frequency were significantly correlated to the rate of temperature changes. However, bursting almost entirely ceased during the steady state of both stimulus protocols. Moreover, the higher rate of burst occurrence was responsible for the initial peak in spike frequency observed during fast temperature drops. In the slow stimulation protocol with a constant rate of temperature drop, bursts were distributed across the entire temperature range, with an average frequency significantly lower than that observed in the fast protocol. These findings strongly suggest that the primary functional role of burst activity is to report the high rate of temperature change. In addition, there was a slight but significant increase in the rate of burst occurrence as the temperature decreased during the descending phase of the slow stimulus. This indicates that bursting may also encode, to some extent, the decrease in absolute temperature. We developed an empirical biophysical model reproducing this repertoire of patterns and proposing basic mechanisms explaining the CIII responses. Our results suggest that the tonic spiking activity contributes to encoding both the rate of temperature change and the end-temperature value, whereas the bursting activity encodes the rate of change in temperature. These findings also shed light on mechanisms underlying the multimodality of sensory cells. 

### 3.1. Multimodality and Features Coding with Patterns

*Drosophila* CIII somatosensory neurons are multimodal, displaying activation in response to noxious cold [9,14] and innocuous mechanical stimuli [5,6]. These different modalities evoke distinct levels of intracellular Ca^2+^ and the distinct behavior of *Drosophila melanogaster* larvae [9]. Noxious cold evokes significantly higher calcium levels in CIII neurons compared to gentle touch [9]. The activation of CIII neurons with noxious cold mediates the bilateral contraction of *Drosophila* larvae, whereas the gentle touch modality is accompanied by head-withdrawal behavior [9]. The gentle touch modality elicits a brief spiking response in CIII neurons with very fast adaptation, where the spiking frequency decreases to zero despite continuing mechanical stimulation [6]. The number of action potentials and the magnitude of calcium response increased with greater mechanical stimulation [6]. Cold-evoked spiking responses of CIII neurons are not well understood. In our previous studies, we demonstrated that CIII neurons exhibit a sustained spiking rate in response to noxious cold stimuli, unlike their response to gentle touch [14]. We observed that their firing rate increases as the final temperature of the stimulus decreases [14]. Moreover, CIII neurons respond with a peak firing rate to fast temperature decrease and suppress activity during temperature increase [14]. Similarly, in polymodal *Drosophila* class IV neurons, which mediate aversive rolling and photo-avoidance behaviors, different modalities evoke distinct patterns of activity [30,31]. Noxious heat (but not other modalities) triggers the occurrence of short bursts, i.e., doublets or sometimes triplets of spikes characterized by very short interspike intervals (groups of ‘unconventional spikes’) followed by long pauses [31,32]. This specific burst–pause pattern has been implicated in triggering robust heat-avoidance rolling behavior of *Drosophila* larvae [31]. The number of unconventional spikes displays a positive correlation with peak amplitudes of Ca^2+^ transients. Thus, pauses and unconventional spikes participate in the coding of the noxious heat stimuli [32]. This noxious heat-specific activity pattern is mediated by coordinated activities of TRP channels (TRPA1 and Painless), L-type voltage-gated Ca^2+^ channels, and Ca^2+^-activated K^+^ (SK) channels [31,32]. Temperature-sensitive neurons are often multimodal and can change their activity pattern under the application of agonists of thermoTRP channels. For example, menthol sensitivity is conserved across mammalians’ and insects’ TRPA1 and TRPM channels [13,44,45,46]. In *Drosophila* larvae, the topical application of menthol evokes aversive rolling behavior [13]. This behavior was mediated by the activation of CIV nociceptor neurons expressing TRPA1 and TRPM channels. Menthol superfusion activates CIV neurons and significantly increases spike frequency compared to the control group [13]. In feline nasal and lingual cold receptors at higher temperatures, above 25 °C, menthol induces bursting or enhances it through an increase in burst frequency [33]. At cold temperatures, menthol suppresses bursting activity [33]. 

Our results show that different information from the same modality can be encoded with distinct activity patterns, namely, information about the rate of temperature change can be encoded with bursting activity, whereas tonic spiking activity can encode both the rate of change and cold-temperature magnitude. Similarly, burst-like activity, which occurs as a transient strong response to temperature change, has been reported in other thermosensory neurons [34,35]. Distinctly, in the hygrothermoreceptor neurons of carabids, it was shown that bursting activity does not convey temporal information about the stimulus but rather encodes its magnitude [36]. 

### 3.2. Roles of Bursting Activity in Responses of Sensory Neurons

In addition to its role in distinguishing sensory signal modalities, bursting activity has been identified to play various distinct functional roles in sensory systems, including mammalian auditory and visual ones. For example, bursts can increase the reliability of information transfer at synapses, and bursting activity mediates orientation and navigation as well as conveys parallel codes that contain different stimulus features, i.e., to function as feature detectors [20,37,38,47,48]. In the present study, we observed not only the initial transient bursts during nociceptive stimulation but also low-frequency, sustained bursting activity in response to mild temperature stimuli. 

Although CIII neurons in a *Drosophila* larva respond to temperature drop similarly to mammalian cold-sensitive neurons, with a peak in the spiking rate, there is a difference in their responses to steady cold temperatures. The majority of *Drosophila* CIII neurons have irregular spiking activity, whereas mammalian cold-sensitive neurons respond with regular patterns that change with cold-temperature magnitude [23,39,40]. For example, cold thermoreceptor neurons have regular spiking activity at resting temperatures of skin (33–34 °C) and bursting activity at lower temperatures. Period of bursting activity, burst duration, average interspike interval inside burst, and the number of spikes per burst increase with temperature decrease [23,39,40]. At low temperatures, such as 10 °C, about 50% of mammalian cold-sensitive fibers exhibited irregular patterns, while the other 50% were either bursting or silent. In *Drosophila* larval CIII neurons, only 2.5% of ddaA and 3.8% of ddaF neurons exhibited an irregular occurrence of bursts at a steady temperature. In contrast, in mammalian cold-sensitive neurons, bursting activity is regular and is described as a regime of activity with temporal properties evolving along with a variation in the temperature parameter within a wide range of values—from about 30 °C to noxious cold [23,39,40]. Thus, *Drosophila* larval CIII neurons and mammalian cold-sensitive neurons encode cold-temperature magnitude in different ways: with rate coding for *Drosophila* and pattern-based coding for mammalians. 

In a variety of sensory systems, bursts were implicated in conveying specific information about stimuli [41]. For example, receptor neurons of the grasshopper auditory system demonstrated a sparse neural code for auditory stimuli, where the number of spikes in a burst encodes information about the amplitude and duration of sound. Burst onset time corresponds to the time of the sound [42]. The functional importance of bursting was shown in the AN2 neuron of cricket *Teleogryllus oceanicus,* which encodes the direction of the sound and predicts an ultrasound-avoidance behavioral response [38]. In electric fish, *Apteronotus leptorhynchus*, the pyramidal cell distinctly encodes behaviorally relevant stimuli with patterns of spiking activity: low-frequency (prey-like) stimuli with bursting and high-frequency (communication) stimuli with isolated spikes [20]. The muscle stretch receptors of the crab *Cancer borealis* encode rapidly and slowly varying stimuli with different activity patterns [43]. Bursting patterns correspond to long, sustained muscle stretches, whereas spiking activity encodes fast stretches. Noxious and innocuous stimuli are also encoded by distinct patterns. For example, neurons in the rostral trigeminal relay nuclei of cats demonstrate characteristic distinct firing patterns under innocuous and noxious mechanical stimulation [49]. Neurons’ responses to the noxious stimulus were frequently bimodal and prolonged, while the non-noxious stimulus elicited a monomodal short response. In addition, a change in the normal activity pattern in sensory neurons to abnormal high-frequency spontaneous activity can be a signature of pathological conditions such as neuropathic or inflammatory pain [50,51,52]. For example, high-frequency spontaneous bursting activity and underlying subthreshold membrane oscillations in sensory neurons are associated with the local inflammation of the DRG and mechanical pain [26,53]. Cells with such high-frequency bursting activity had an upregulated expression of Na_v_1.6. Erythromelalgia, autosomal dominant painful neuropathy, is characterized by bursting activity in DRG sensory neurons [54]. Such a disorder is caused by a mutation in Na_v_1.7, which reduces the threshold for action potential and leads to high-frequency activity in DRG neurons [54]. 

### 3.3. Dynamics of a thermoTRP Current Navigates Spiking Responses to the Temperature Change

To explain the mechanism of cold-temperature coding, we developed an empirical computational model of cold-sensitive CIII neurons. The model is informed by transcriptomic data from larval CIII neurons [9] and includes a pattern-generating subsystem and a thermotransduction subsystem represented by a thermoTRP current [14] (level-II model). The conductance of the TRP current is activated by low temperature and inactivated by intracellular Ca^2+^ concentration. It also is permeable for Ca^2+^, which provides negative feedback to the TRP conductance and activates SK and BK K^+^ currents. We developed the model in two empirical steps. First, by adjusting mainly kinetic parameters of voltage-gated Ca^2+^, fast Na^+^, and fast K^+^ currents of the pattern-generating subsystem (level-I model) with the conductance of the thermoTRP current kept constant (*G_LTRP_*), we found a parameter set that captured the repertoire of all basic CIII activity patterns observed in the experimental data. We mapped the basic steady-state activity patterns, i.e., silence, tonic spiking, period-2 spiking, and bursting, on a diagram with two parameters: *G_LTRP_* and temperature. We found that the silent and tonic spiking regimes occupy the largest areas on the diagram. The domain of the silent regime has the largest range of temperature at 0 nS conductance of the TRP current and shrinks as *G_LTRP_* is increased. Conversely, the tonic spiking regime occupies the largest range of temperature at the high end of *G_LTRP_* and shrinks towards smaller values. In a certain relatively small range of *G_LTRP_*, we found a peninsula-like range of temperatures in which the subsystem generated bursting activity. Second, this map was handy in the dissection of the contributions of the thermoTRP current and the pattern-generating subsystem. We projected the instantaneous value of the conductance of the thermoTRP current in the full model (level-II model). We found that TRP activation and inactivation by defining instantaneous TRP conductance determines the response of the model to temperature change and could recapitulate and explain the key experimental observations. In this way, we can observe different temperature coding mechanisms depending on the dynamics of the TRP current: coding of the rate of temperature change with bursting and tonic spiking; and coding of steady-state temperature magnitude with spiking rate. The variability of parameters describing the kinetics of the TRP current produced a variability of paths on the map, which allows us to relate these parameters to characteristics like the presence of bursting in responses and produce similar results to the variability of CIII responses found in experimental data between different cells. Thus, dynamics of TRP conductance shape the path on the map of activities and produce various types of CIII cold-evoked responses to the fast temperature protocol: a bursting pattern during temperature change when the neuron response path crosses the region of bursting or passes in its vicinity, and tonic spiking activity at a steady-state noxious cold temperature; bursting activity during both temperature change and at a steady temperature; and pure tonic spiking activity with or without a peak in the spiking rate (Figure 6, Figure 7 and Figure 8). All these model results recapitulate the electrophysiological data obtained from the CIII neurons. In addition, the model demonstrated that intra-burst frequency depends on the rate of temperature change but does not depend on cold-temperature magnitude, which is also consistent with experimental results. Together, the results indicate that the magnitude of temperature decrease could be encoded by tonic spiking activity, whereas the rate of temperature change could be encoded by either bursting or tonic spiking.

Our study centered on the role of TRP channels in the cold-evoked activity patterns of CIII neurons, as these channels have been implicated in cold-temperature sensation [9]. However, the contribution of various non-TRP currents to activity patterns can be significantly modified at distinct temperature levels [55,56]. It is worth highlighting that several biophysical models of mammalian cold receptors have suggested that cold thermosensation can arise without TRP channels. This was attributed to temperature-dependent factors scaling the time constants and maximal conductances for both inward and outward non-TRP currents [57,58,59,60]. Consequently, in these models, excitability and neural activity patterns differ at various temperatures and could code temperature. In our model, thermotransduction is primarily driven by the dynamics of a TRP current. While the model does account for the temperature-dependent scaling factors of non-TRP channels, it is essential to note that without the TRP current, the neuron remains quiescent throughout the entire temperature range considered. 

Our research on the cellular mechanisms underlying noxious cold-stimuli coding provides an understanding of how primary sensory CIII neurons of *Drosophila* larvae transduce features of thermal stimuli (rate of temperature change and cold-temperature magnitude) into specific patterns of electrical activity. Considering that CIII neurons express different types of TRP channels involved in cold sensation, including PKD2, Trpm, and TRPA1, each of these channels could differently impact the characteristics of cold-evoked CIII responses. Our CIII model demonstrates the fundamental principles of how TRP currents with different dynamics can navigate a neuron’s response pattern. Particularly, we showed how the dynamics of TRP currents can modulate the cell’s response to a noxious cold stimulus, making it spike or burst. A deeper understanding of the cellular mechanisms of pattern generation during cold sensation, as well as the dynamics of different TRP channels co-expressed in sensory neurons, could potentially lead to new therapeutic strategies for the treatment of sensory neuropathies. 

## 4. Materials and Methods

### 4.1. Animals

The larvae of *Drosophila melanogaster* emerged from eggs and were raised on standard cornmeal/molasses/agar diet in a 12:12 h light–dark cycle at room temperature (21–24 °C) [11,15]. To visualize CIII neurons under fluorescence microscopy, we used third instar larvae (96–120 h after egg laying) expressing *UAS-mCD8::GFP* (Bloomington *Drosophila* Stock Center #5130, Indiana University in Bloomington, IN, USA) under the control of *GAL4^19–12^*, which was generously gifted by Dr. Y-N Jan [61].

### 4.2. Electrophysiology and Statistical Analysis

The third instar *Drosophila* larvae were dissected as described previously [14]. Briefly, the ventral body wall was cut longitudinally, and the body wall was spread out and pinned to the bottom of the experimental dish. Dorsal longitudinal muscles were carefully removed with fine scissors and a tungsten needle to expose the dorsal cluster of the sensory neurons. The dish was constantly superfused with gravity-dripped HL3 saline [62]. 

Extracellular recordings were made from either ddaA or ddaF with a pipette (tip diameter, 5–10 µm) connected to the headstage of a patch-clamp amplifier (AxoPatch 200 B or MultiClamp 700 A, Molecular Devices, San Jose, CA, USA) set to voltage-clamp mode held at 0 mV. The high-frequency noise of the current signal was filtered out with a 3 kHz low-pass filter. The output signals of the amplifier were digitized at a sampling frequency of 10 kHz using an A/D converter (Micro1401-4, Cambridge Electronic Design, Cambridge, UK) and transferred to a laptop computer running Windows 10 (Microsoft, Redmont, WA, USA) with Spike2 software v. 8 (Cambridge Electronic Design, Cambridge, UK). The saline temperature was continuously recorded by a BAT-12 Microprobe Thermometer (Physitemp, Clifton, NJ, USA). The temperature probe was placed adjacent to the fillet preparation, and the readings were sent to Micro1401.

To detect spikes in the saved data, we set the amplitude threshold in the Spike2 software function. The instantaneous frequency was directly determined from spike intervals (Figure 2A); the spiking rate (spikes/s) was calculated as an average over a fixed time window of 30 s (Figure 2C,D), 2 s (Figure 7), or 20 s (Figure 8). A burst was defined as a series of three or more spikes with inter-spike intervals of 0.2 s or less, while those with larger spike intervals were defined as tonic spikes (Figure 2). When there were more than six spikes at intervals of 0.2 s or less, any peak in the spike interval was considered a break between bursts. To determine the relationship between the spiking rate and temperature in the slow stimulation protocol, we plotted the average of the spiking rate in a 2 °C temperature bin (Figure 8).

Statistical comparisons were conducted using SigmaPlot ver. 12.5 (Jandel Scientific, San Rafael, CA, USA) for Student’s *t*-test (two-tailed) and one-way repeated measures (RM) ANOVA, followed by pairwise multiple comparison procedures using the Holm–Sidak method. The assumption of normality of the data structure was assessed using the Shapiro–Wilk test for all tests. When the assumption of normality was not met, Friedman RM ANOVA on ranks, the Mann–Whitney Rank Sum test, or the Wilcoxon Signed Rank Sum test was employed. The results are presented as the mean ± standard error or standard deviation, as specified in the text.

### 4.3. Cold-Temperature Stimulation

We used two different methods to apply the cold stimulus while recording spiking activity from a CIII neuron, as described previously [14]. One approach is to switch perfusates from the saline held at room temperature to one pre-chilled to the desired temperature (fast stimulation protocol). The other method is directly controlling the perfusate by sending a command voltage waveform to the temperature control device (slow stimulation protocol). In the fast stimulation protocol, the cooler temperature is set to the target temperature (20 °C, 15 °C, or 10 °C) in advance to chill the saline to the desired temperature before delivery. This protocol can change the temperature of the saline in the dish at a rate of −2 to −6 °C/s. In the slow stimulation protocol, the temperature stimulus was applied by directly changing the perfusate temperature at the rate of −0.12 °C/s through the in-line cooler, without turning the valve to switch the saline path. The waveform of the temperature change is created by the graphical sequence editor function in Spike2 software. The command output was directly fed to the temperature controller (CL-100) as a voltage signal from the DAC output of the data acquisition interface.

### 4.4. Computational Model

We developed a biophysical Hodgkin–Huxley-type model of the *Drosophila* larva cold-sensitive CIII neuron. A full description of the CIII currents is given in our previous work [14]. In the present study, we made a few modifications and improved our model. The suggested ionic currents used in our model were based on transcriptomic data of ion channels’ expression in CIII neurons [9]: voltage-gated Na^+^ current, *I_Na_*—encoded by the *para* gene; delayed rectifier potassium current, I_K_ encoded by the *shab* gene; voltage-gated N-type calcium current, *I_Ca_*; Ca^2+^ activated potassium channels: small conductance, *I_SK_*, and big conductance, *I_BK_*, encoded by SK and *slowpoke* genes, respectively; leak current, *I_L_*; and TRP current, *I_TRP_* [14].

To perform a detailed analysis of electrical activity regimes, we developed a CIII model with two levels of complexity regarding the TRP current. Using the level-I model, where the TRP current is represented by a leak current component with constant conductance (*G_LTRP_*), we investigated the steady-state electrical activity regimes of a CIII neuron model. In the level-II model, the conductance of the TRP current was dynamic and was governed by activation and inactivation. With the level-I, state-dependent model, we investigated the model response to changing temperature stimulation. 

Custom MATLAB scripts were utilized to perform simulations and data analyses. For solving the system of differential equations that described the CIII neuron model, we employed the variable-step integration method with backward differentiation formulas. We set the absolute tolerance to 10^−9^ and the relative tolerance to 10^−8^ and used the MATLAB function ode15 s to obtain a numerical solution.

#### 4.4.1. Level-I Model

In the level-I model, the TRP current is phenomenological and represented as a nonspecific leak current, *I_LTRP_*, permeable to Na^+^, K^+^, and Ca^2+^, with a reversal potential close to zero. 

The dynamics of the electrical activity of the CIII model are described by the equation:

(1) $$\frac{dV_{m}}{dt}=-\left[I_{Na}+I_{K}+I_{Ca}+I_{BK}+I_{SK}+I_{L}+I_{LTRP}\right]/C_{m}$$

where *V_m_* is a membrane potential in mV, *t* is time in s, *C_m_* is a total neural membrane capacitance, *C_m_* = 0.01 nF. The currents in the model are described by the following equations:

$$I_{Na}=\rho \left(T\right)\cdot \bar{G_{Na}}\cdot m_{Na}{}^{3}\cdot h_{Na}\cdot \left[V_{m}-E_{Na}\right],$$

$$I_{K}=\rho \left(T\right)\cdot \bar{G_{K}}\cdot m_{K}{}^{4}\cdot \left[V_{m}-E_{K}\right],$$

$$I_{Ca}=\rho \left(T\right)\cdot \bar{G_{Ca}}\cdot m_{Ca}\cdot h_{Ca}\cdot \left[V_{m}-E_{Ca}\right],$$

$$I_{BK}=\rho \left(T\right)\cdot \bar{G_{BK}}\cdot f_{CaBK}\cdot m_{BK}{}^{4}\cdot \left[V_{m}-E_{K}\right],$$

$$I_{SK}=\rho \left(T\right)\cdot \bar{G_{SK}}\cdot f_{SK}\cdot \left[V_{m}-E_{K}\right],$$

$$I_{L}=\rho \left(T\right)\cdot G_{L}\cdot \left[V_{m}-E_{L}\right],$$

$$I_{LTRP}=G_{LTRP}\cdot \left[V_{m}-E_{TRP}\right],$$

where $\bar{G_{i}}$—is the maximal conductance in nS, $E_{i}$ is the reversal potential in mV, $m_{i}$ and $h_{i}$ are dimensionless activation and inactivation gating variables of current *i*, with *i* ϵ {*Na*, *K*, *Ca*, *BK*, *SK*, *TRP*} respectively; ρ(*T*)—temperature-dependent scaling factor for the maximal conductances $\rho \left(T\right)=1.3^{\left(T-T_{0}\right)/10},$ where *T* is a temperature in Kelvin (K), *T_0_* = 298.15 K is a reference temperature [63]. We used temperature in Kelvin for the parameters of our biophysical model, while in figures, for consistency with experimental data, modeling results are presented in °C.

Gating variables are described with the following equations:

$$\frac{dy_{i}}{dt}=\varphi \left(T\right)\cdot \frac{y_{\infty i}\left(V_{m}\right)-y_{i}}{\tau_{i}\left(V_{m}\right)},$$

$$\frac{df_{i}}{dt}=\varphi \left(T\right)\cdot \frac{f_{\infty i}\left(Ca\right)-f_{i}}{\tau_{i}\left(Ca\right)},$$

where $y_{\infty i}$ is a steady-state activation/inactivation for the current *i* ϵ {Na, K, Ca, *BK*}; $f_{\infty i}$ is a steady-state activation *SK* and *BK* currents. 

φ(T) is a temperature-dependent scaling factor for activation and inactivation kinetics, $\varphi \left(T\right)=3^{\left(T-T_{0}\right)/10}$ [63].

Steady-state activation for Na^+^, K^+^, Ca^2+^ currents is described with equation:

$$m_{i\infty }\left(V_{m}\right)=\frac{1}{1+e^{\frac{-\left(V_{m}-V_{mi}\right)}{K_{mi}}}};$$

Steady-state inactivation for Na^+^ and Ca^2+^ currents are defined as:

$$h_{i\infty }\left(V_{m}\right)=\frac{1}{1+e^{\frac{\left(V_{m}-V_{hi}\right)}{K_{hi}}}}.$$

The steady-state activation of *BK* current consists of the Ca^2+^-dependent part: $f_{CaBK}\left(Ca_{i}\right)=\frac{1}{1+{\left(\frac{Ca_{BK}}{Ca_{i}}\right)}^{n_{BK}}}$ and the voltage-dependent part: $m_{BK\infty }\left(V_{m}\right)=\frac{1}{1+e^{\frac{-\left(V_{m}+28.3\right)}{30}}}$.

The steady-state activation of *SK* current is Ca^2+^-dependent and obeys the following equation: $f_{SK\infty }\left(Ca_{i}\right)=\frac{1}{1+{\left(\frac{Ca_{SK}}{Ca_{i}}\right)}^{n_{SK}}}$.

In our model, BK, K^+^ and Na^+^ currents have voltage-dependent kinetics that are described with following equations:

$$\tau_{mBK}\left(V_{m}\right)=\frac{-0.1502}{1+e^{\frac{-\left(V_{m}+46\right)}{22.7}}}+0.1806;$$

$$\tau_{hNa}\left(V_{m}\right)=(\frac{4.5}{\cosh\left(\frac{V_{m}+V_{hNa}}{3K_{hNa}}\right)}\;+0.75)/1000;$$

$$\tau_{mK}\left(V_{m}\right)=\left(\frac{5}{\cos h\left(\frac{V_{m}+V_{mK}}{2K_{mK}}\right)}+0.75\right)/1000;$$

Ca^2+^ dynamics are described by the differential equation:

$$\frac{dCa_{i}}{dt}=-\frac{I_{Ca}+I_{TRP_{Ca}}}{F\cdot z\cdot Vol}-k\cdot \left(Ca_{i}-Ca_{min}\right)$$

where $I_{TRP_{Ca}}$ is a Ca^2+^ component of the *TRP* current. It is calculated as:

$$I_{TRP_{Ca}}=\bar{G_{TRP}}\cdot \frac{P_{Ca}}{P_{K}P_{Na}+P_{Ca}}\left[V_{m}-E_{Ca}\right].$$

The reversal potential for the *TRP* current takes into account changes in the intracellular Ca^2+^ concentration and is calculated as:

$$E_{TRP}=\frac{P_{K}E_{K}+P_{Na}E_{Na}+P_{Ca}E_{Ca}^{*}}{P_{K}+P_{Na}+P_{Ca}},$$

where $P_{K},P_{Na},P_{Ca}$ are relative permeabilities for corresponding ions. Relative permeabilities for K^+^ and Ca^2+^ were constant—1 and 0.4, respectively—whereas permeability for Na^+^ was calculated considering zero reversal potential for the taken reversal potentials:

$$P_{Na}=-\left(P_{K}E_{K}+P_{Ca}E_{Ca}^{*}\right)/E_{Na}$$

where $E_{K},E_{Na},E_{Ca}^{*}$ are equal to −75 mV, 65 mV, 120 mV, correspondingly.

The Ca^2+^ reversal potential, $E_{Ca}$, dynamically changes in time and is calculated as the Nernst potential using $Ca_{i}$ and assuming that the external Ca^2+^ concentration ($Ca_{e}$) is constant and equal to 2 mM: $E_{Ca}=1000\frac{RT}{zF}ln\frac{Ca_{e}}{Ca_{i}}$, where *R, F,* T are the gas constant, Faraday’s constant, and temperature in Kelvin, respectively.

The reversal potential for a leak current, *E_L_*, is −75 mV.

We used same parameters for the model as in [14], except for $\bar{G_{L}}$ and $K_{hCa},\;\mathrm{which}\;\mathrm{are}\;\mathrm{modified}$: $\bar{G_{Na}}=80\;\mathrm{nS}$, $\bar{G_{K}}=140\;\mathrm{nS}$,$\;\bar{G_{Ca}}=3.5\;\mathrm{nS}$, $\bar{G_{BK}}$ = 6 nS, $\bar{G_{SK}}$ = 0.31 nS, $\bar{G_{L}}=0.25\;\mathrm{nS}$, $n_{BK}=3$, $Ca_{BK}$ = 1700 nM, $Ca_{SK}=800\;\mathrm{nM}$, $n_{SK}=3$, $V_{mNa}=-24.7\;\mathrm{mV}$, $K_{mNa}=3.4\;\mathrm{mV}$, $V_{hNa}=-41.2\;\mathrm{mV}$, $K_{hNa}=4.2\;\mathrm{mV}$, $V_{mK}=12\;\mathrm{mV}$, $K_{mK}=7\;\mathrm{mV}$, $V_{mCa}=-23\;\mathrm{mV}$, $K_{mCa}=6.5\;\mathrm{mV}$, $V_{hCa}=-59\;\mathrm{mV}$, $K_{hCa}=12\;\mathrm{mV}$, *F* = 96,485.35 × 10^−9^ C/nmol, *R* = 8.31 × 10^−9^ J/(nmol×K), *C_m_* = 0.01 nF, *z* = 2, *Vol* = 0.2 pL, *k* = 403 s^−1^, $Ca_{min}$ = 50 nM, *[Ca^2+^]_e_* = 2 × 10^6^ nM, $\tau_{mSK}=0.04\;s$, $\tau_{mCa}=0.0035\;s$, $\tau_{hCa}=0.095\;s$, $\tau_{mNa}$ = 0.0001 s.

For creating color maps, *G_LTRP_* was changed from 0 to 1 nS with a step of 0.02 nS (rows); and steady-state temperature was changed from 24 °C to 4 °C with a step of −0.5 °C (columns). We made a pre-integration for 100 s at room temperature, and then, at every change of *G_LTRP_* or temperature, we made two integrations: the first integration was 60 s, after we took the last point from the first integration as the initial condition for the second integration and integrated for 40 s. During these 40 s, we conducted the analysis of patterns, spike frequency, intracellular Ca^2+^ levels, $Ca_{i}$, and average number of spikes in the burst (Figure 1). For this analysis, we wrote a custom MATLAB script. In the tonic spiking area, we calculated mean tonic spiking frequency, and for the bursting area, we calculated the mean frequency inside bursts (Figure 1E). 

#### 4.4.2. Level-II Model (Full Model)

For level-II model, we upgraded the level-I model with temperature activation, $m_{TRP}$, and the Ca^2+^-dependent inactivation, $h_{TRP}$, of the TRP current, *I_TRP_*. For the level-II model, *I_TRP_* current is used in Equation (1) instead of *I_LTRP_.*

$$I_{TRP}=\bar{G_{TRP}}\cdot m_{TRP}\cdot h_{TRP}\cdot \left[V_{m}-E_{TRP}\right]$$

Gating variables for the *TRP* current are described with the following equations:

$$\frac{dm_{TRP}}{dt}=\frac{m_{TRP\infty }\left(T\right)-m_{TRP}}{\tau_{mTRP}},$$

$$\frac{dh_{TRP}}{dt}=\frac{h_{TRP\infty }\left(T\right)-h_{TRP}}{\tau_{hTRP}}.$$

We applied the following expression for steady-state *TRP* activation:

$$m_{TRP\infty }\left(T\right)=\frac{B}{1+e^{-A\left(T-T_{h}\right)}},$$

where *A*—the steepness of temperature dependence of TRP activation, *B*—activation scaling factor, *T_h_*—the temperature of the half-activation in the Boltzmann function in *K*, *T*—the temperature in *K*.

The steady-state inactivation of the *TRP* current is:

$$h_{TRP\infty }\left(Ca_{i}\right)=1-\frac{Ca_{i}^{N}}{Ca_{h}^{N}+Ca_{i}^{N}}$$

where $Ca_{i}$—intracellular Ca^2+^ concentration, $Ca_{h}$—the half inactivation Ca^2+^ concentration, *N*—the Hill coefficient. The concentration of cytosolic Ca^2+^ is a crucial factor in the process of TRP inactivation, which involves several sub-processes and stages that are not yet fully understood and quantitatively described. We focused our investigation on the phenomena and variables that we hypothesize to be critical or dominant. When the cytosolic Ca^2+^ concentration increases, downstream pathways are activated that lead to the desensitization of many TRP channels. The process of desensitization can occur via kinases, phosphatases, phospholipases, or calmodulin [64,65]. In our model, we used a general expression for TRP inactivation that reflects the Ca^2+^-dependent modulation of the TRP current, which can be induced by these pathways.

$I_{TRP_{Ca}}$, a Ca^2+^ component of the TRP current, is calculated as

$$I_{TRP_{Ca}}=\bar{G_{TRP}}\cdot m_{TRP}\cdot h_{TRP}\cdot \frac{P_{Ca}}{P_{K}+P_{Na}+P_{Ca}}\left[V_{m}-E_{Ca}\right]$$

The parameters for the *TRP* current, used in Figure 7, Figure 8 and Figure 9, are: $\bar{G_{TRP}}$ = 1.2 nS, T_h_ = 290.15 K, A = 1 K^−1^, *N* = 2, *Ca_h_* = 700 nM, $\tau_{hTRP}$ = 10 s, $\tau_{mTRP}$ = 0.002 s, B = 1.

For our level-II model, we made two integrations: the first integration was 100 s at room temperature (the initial room temperature from the temperature protocols provided). Then, we took the last point from the first integration as the initial condition for the second integration. The time of the second integration was the same as the time of the corresponding temperature protocol.

Using temperature traces recorded from the microprobe thermometer, we applied thermal stimulation to our model, following the same temperature protocols as those used in experimental recordings. Additionally, we administered trapezoid stimulation temperature protocols with two variations. In the first case, the temperature was held at 24 °C for 30 s and then gradually decreased at different rates (ranging from 0.1 to 5.5 °C/s with an increment of 0.1 °C/s per trial) until it reached a target value of 10 °C, where it was maintained for another 30 s. The temperature was then gradually raised to 24 °C at the same rate and held constant for 30 s. In the second case, the temperature was held at 24 °C for 30 s and then gradually decreased at a constant rate of 3 °C/s until it reached different target temperatures (ranging from 20 °C to 6 °C with a decrement of 0.5 °C per trial). Once it reached the target temperature, it was held constant for 30 s before gradually increasing to 24 °C at the same rate. The temperature was then held constant at this level for 30 s. 

For the investigation of the effect of TRP current parameters on model activity patterns, we used experimentally obtained sets of parameters assessed by the curve-fitting of spiking rates, presented in our previous work [14]. These parameter sets are given in Table S1, Group I in [14]. As the temperature protocol for the model, we used 10 °C fast stimulation temperature traces, with small variations in temperature stimulations from experiment to experiment applied to biological neurons. We obtained color maps of spiking rates for every fixed temperature trace for different parameter sets (Figure S3). Electrical activity patterns were analyzed for 22 × 22 model traces. 

## Data Availability

MATLAB scripts developed in this study can be found at https://senselab.med.yale.edu/ModelDB/.

## Supplementary Materials

The supporting information can be downloaded at: https://www.mdpi.com/article/10.3390/ijms241914638/s1.
