# Supplementary material for: Cold-Temperature Coding with Bursting and Spiking Based on TRP Channel Dynamics in Drosophila Larva Sensory Neurons
Source: Int J Mol Sci. 2023 Sep 27;24(19):14638. doi: 10.3390/ijms241914638 (PMC10572325; doi:10.3390/ijms241914638)
Supplement: Supplementary file 1 [file ijms-24-14638-s001.zip › ijms-2463353-supplementary.pdf]

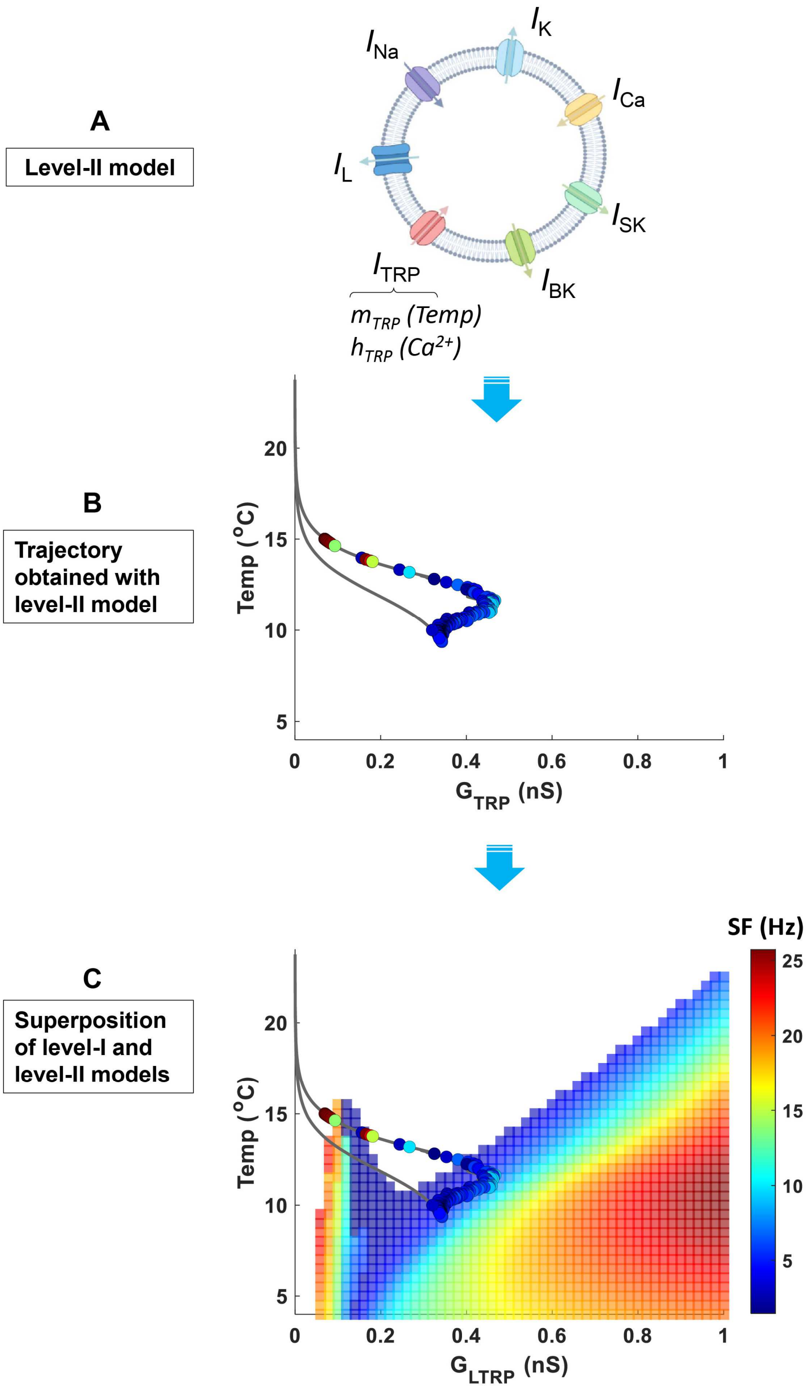

Figure S1. **Projection of trajectory for instantaneous TRP conductance obtained with a level-II model (a full CIII model) onto the 2-parameter map of the level-I model allows us to investigate CIII model activity patterns.** (**A**) Schematic representation of level-II CIII model with temperature-dependent activation and Ca^{2+}-dependent inactivation of TRP current. (**B**) Trajectory of TRP conductance obtained with level-II model. (**C**) Superimposed trajectory of the level-II model onto color map obtained with level-I model. Circles on the trajectory indicate individual spikes. Colors inside the circle encode mean spike frequency (SF).

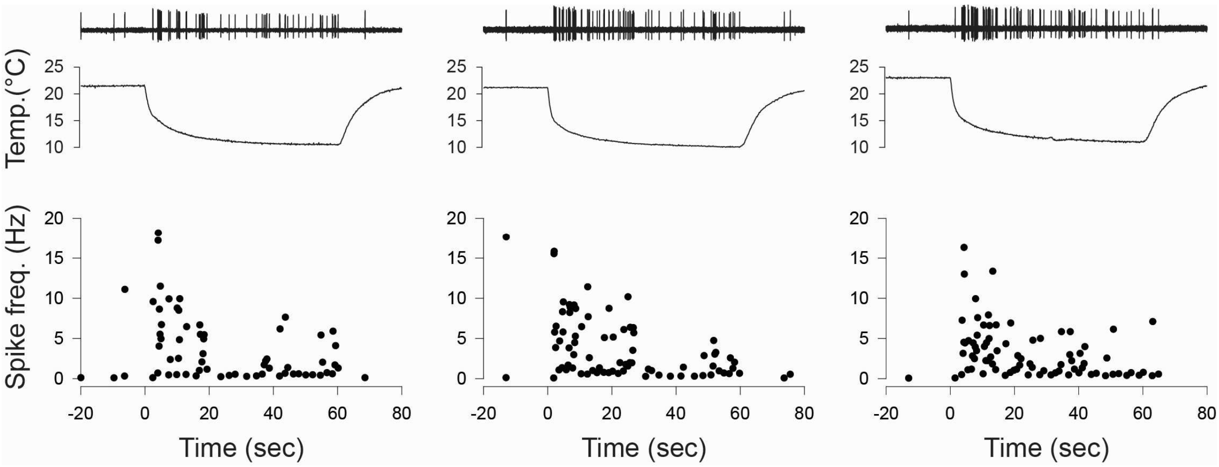

Figure S2. Repeated stimulation of the same neuron did not induce significant changes in its spiking property. Representative data of spiking activity (top traces) of CIII neurons during three repetitions of a fast stimulation protocol with temperature reduced to 10°C (middle trace). Stimulation intervals were at least 2 min apart. The bottom graphs show plots of instantaneous spike frequencies.

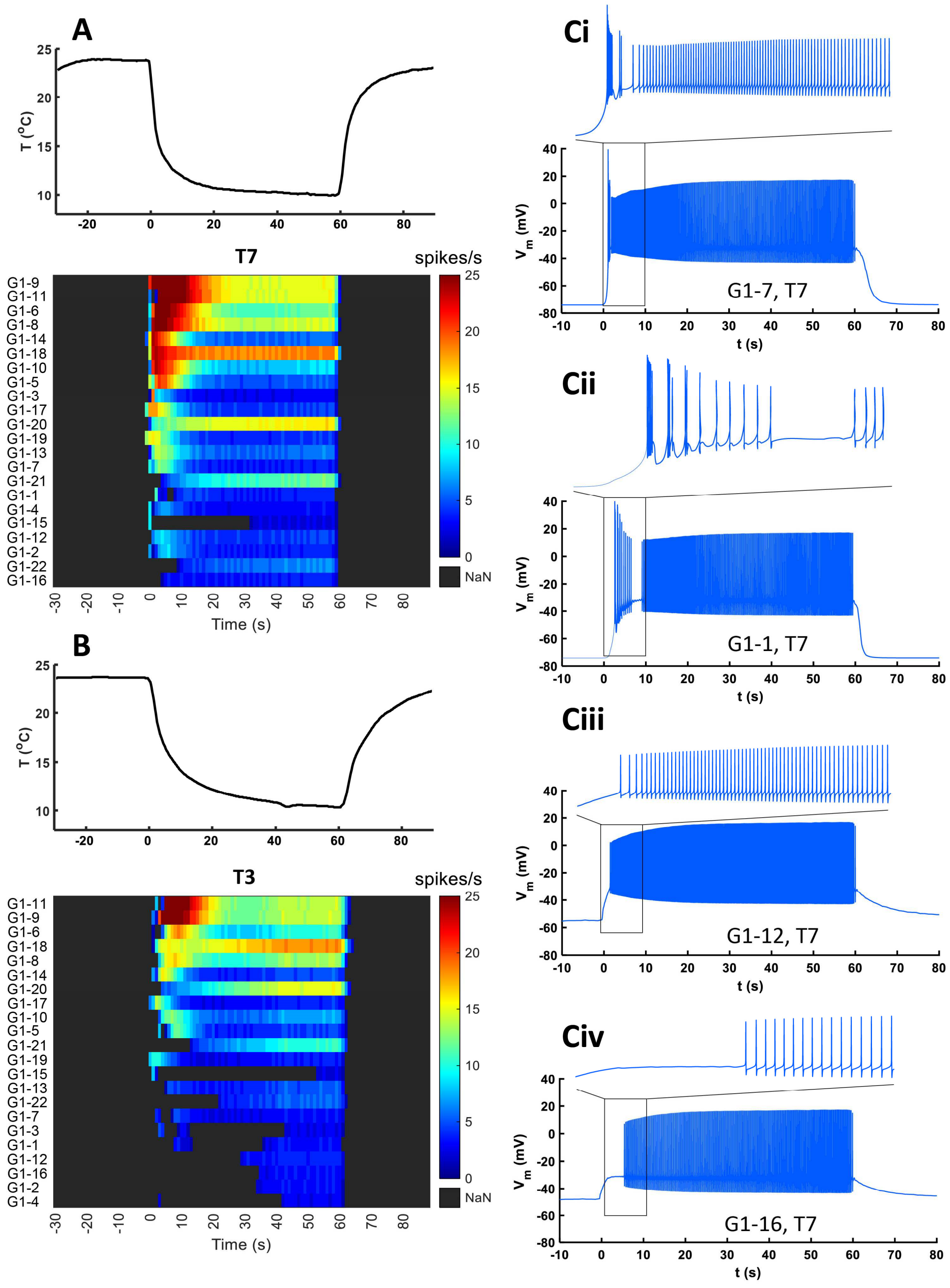

Figure S3. **Different parameters sets obtained from curve fitting of experimental temperature-response curves and variability in temperature protocols affect CIII activity patterns: bursting tonic spiking**. (A-B) Color maps coding spiking responses (x axis) of the CIII model to two representative experimental fast temperature stimulation traces, T3 and T7. The color maps use 22 TRP parameter sets, G1-1 – G1-22, (y axis) obtained from curve fitting of experimental temperature-response curves [14]. (Ci-Ciii) four representative electrical responses from A that show spiking or bursting responses depending on parameters set. In all color maps, the average frequency was calculated in the 1-s bin window.
